# Intermittent fasting ameliorates MAFLD by downregulating Lrg1: insights from bulk RNA sequencing and functional verification

**DOI:** 10.3389/fendo.2026.1754251

**Published:** 2026-02-16

**Authors:** Huafeng Chen, Shilin Zhang, Wenqiang Xie, Jie Shen, Hua Liang

**Affiliations:** 1 Department of Endocrinology and Metabolism, The Eighth Affiliated Hospital, Southern Medical University (The First People’s Hospital of Shunde, Foshan), Foshan, Guangdong, China; 2 Medical Research Center, The Eighth Affiliated Hospital, Southern Medical University (The First People’s Hospital of Shunde, Foshan), Foshan, Guangdong, China

**Keywords:** fatty acid metabolism, inflammation, intermittent fasting, LRG1, MAFLD

## Abstract

**Background:**

The global prevalence of metabolic dysfunction-associated fatty liver disease (MAFLD) is increasing annually, significantly impairing patients’ quality of life. Given the limitations of existing treatments, this study aims to investigate the effects of intermittent fasting (IF) on MAFLD and its underlying mechanisms.

**Methods:**

The liver tissues of four groups of mice were analyzed by bulk RNA sequencing: normal ad libitum diet (CD group), normal IF (iCD group), high-fat ad libitum diet (HFD group) and high-fat IF group (iHFD group). Differentially expressed genes (DEGs) were identified, followed by enrichment analyses including Gene Ontology (GO), Kyoto Encyclopedia of Genes and Genomes (KEGG), and Gene Set Enrichment Analysis (GSEA). Weighted gene co-expression network analysis (WGCNA) was used to identify related modules. The most highly correlated module genes were intersected with DEGs and analyzed by protein–protein interaction(PPI) network to identify key genes. The key genes were preliminarily verified by RT-qPCR. The function of the key gene was further verified by *in vitro* and *in vivo* experiments.

**Results:**

IF significantly improved metabolic abnormalities and hepatic lipid deposition in MAFLD mice. A total of 331 DEGs were identified between the HFD and CD group, 379 DEGs between the iHFD and HFD group, and 142 DEGs were found to be common to both comparisons. Enrichment analysis showed that DEGs were mainly enriched in pathways related to fatty acid metabolism and inflammatory responses. WGCNA identified red and blue modules are most strongly correlated with MAFLD traits. After intersecting with DEGs, 32 genes were obtained. Based on PPI network analysis, we identified five key genes. After knocking down one of the key genes, Lrg1, *in vitro* and *in vivo*, we confirmed that Lrg1 may promote the expression of lipogenic genes such as Srebf1, Scd1, and Fasn via the PI3K-AKT pathway, thereby accelerating MAFLD progression.

**Conclusions:**

Transcriptome analysis elucidated the potential mechanism by which intermittent fasting improves MAFLD, highlighting the important role of fatty acid metabolism and inflammatory responses. Several key genes regulating MAFLD through IF were identified. Knocking down the key gene Lrg1 inhibited the expression of lipogenic genes and effectively slowed MAFLD progression.

## 1 Introduction

MAFLD has become the most prevalent liver disease globally and a significant public health issue (1). It may progress to liver fibrosis, cirrhosis and even hepatocellular carcinoma (2). Currently, the primary treatment approaches for MAFLD include lifestyle interventions and pharmacotherapy. However, these strategies usually have limitations, including variable treatment responses and individual patient differences (3, 4). Therefore, there is an urgent need for innovative treatment modalities and mechanisms to enhance MAFLD management.

In recent years, IF has gained increasing attention as an emerging dietary intervention model due to its metabolic benefits. Alternate-day fasting (ADF) is the most popular and representative IF regimen. Existing studies have shown that IF exerts protective effects against the occurrence and development of MAFLD by regulating inflammation and lipid metabolism (5).

IF significantly promoted weight loss, improved glucose tolerance, insulin sensitivity, dyslipidemia in mice, and slowed the progression of MAFLD (6–8). It has also been reported that IF down-regulates the expression of peroxisome proliferator activated receptor gamma (PPAR-γ) and cytokines in the liver of obese mice induced by HFD, activates AMP-activated protein kinase (AMPK)/unc-51 like kinase 1 (ULK1) signaling, inhibits the phosphorylation of mechanistic target of rapamycin kinase (mTOR), and inhibits lipid accumulation and inflammatory reaction in white adipose tissue (9). Recent clinical research evidence suggests that IF significantly reduces weight, fat mass, waist circumference, alanine aminotransferase, and improves insulin sensitivity in patients with MAFLD (10). The metabolic benefits of intermittent fasting are multifaceted, including weight loss, improvement of blood sugar and insulin resistance, reduction of oxidative stress, reduction of blood lipid and reduction of systemic inflammatory markers. Its potential mechanism of action is also diverse, including regulating circadian rhythm, adipose tissue and adipokines, intestinal microbiota and autophagy (11). However, The precise transcriptional networks through which IF ameliorates MAFLD and the key mediators coordinating these benefits remain unclear.

In this study, the mechanism of IF in MAFLD was preliminarily analyzed by transcriptome analysis, animal model and cell experiment, including regulating lipid metabolism and inflammatory reactions. Key genes were identified, and the role of the key gene leucine-rich alpha-2-glycoprotein 1 (Lrg1) in MAFLD was verified, which provides theoretical foundation for future research and clinical applications.

## 2 Materials and methods

### 2.1 Animal model construction and intervention scheme

Male C57BL/6 6-week-old mice were purchased from Guangdong Zhiyuan Biomedical Technology Co., Ltd. Animals were kept in SPF environment of the Eighth Affiliated Hospital of Southern Medical University. Temperature and humidity were controlled under a 12-hour light-dark cycle. They were randomly divided into four groups: CD, iCD, HFD and iHFD, n=8. The IF group followed an alternate-day fasting regimen, with fasting or feeding occurring daily at 9 a.m. Food intake was recorded daily, body weight was measured weekly, and fasting blood glucose levels were measured every two weeks during the 12-week intervention.

32 mice in animal experiment 2 were randomly divided into two groups: HFD and iHFD groups. Eight weeks after intervention, mice received tail vein injections of either an Lrg1-knockdown adeno-associated virus (AAV) or a control AAV. This resulted in four groups: high-fat ad libitum diet control (HFD+AAV-Ctrl), high-fat ad libitum diet Lrg1-knockdown (HFD+AAV-shLrg1), high-fat IF control (iHFD+AAV-Ctrl), and high-fat IF Lrg1-knockdown (iHFD+AAV-shLrg1) groups, n=8. They continued their diets for an additional 4 weeks. After completion of all experiments, mice were fasted for 12 h. Mice were anesthetized with 1.25% 2,2,2-tribromoethanol (30 μl/g) by intraperitoneal injection and blood was collected by enucleating the eyeballs. The 2,2,2-Tribromoethanol was purchased from Macklin (catalog number T708333). Mice were then euthanized by cervical dislocation. This study was approved by The Institutional of Animal Care Committee And Animal Ethics Committee of Shunde Hospital, Southern Medical University (The First People’s Hospital of Shunde Foshan) (Approval Number: SDYY-LH-12-2404-010).

### 2.2 Cell model construction and intervention scheme

Alpha Mouse Liver 12 (AML12) cells were purchased from the cell bank of Sevier Biotechnology Co., Ltd. They were cultured in DMEM/F12 medium supplemented with 10% fetal bovine serum, 1% P/S, 1% ITS liquid culture supplement, and 40 ng/mL dexamethasone. The cells were cultured at 37°C with 5% CO_2_. Lrg1 expression was knocked down in AML12 cells by transfecting siRNA (Sense: ACAAGAUGUUCUCACAGAACG, Antisense: UUCUGUGAGAACAUCUUGUUU). Sodium palmitate (PA) was used to induce lipid accumulation in AML12 cells. Based on our previous study, the PA concentration was set at 0.4 mM for 24 hours (12).

### 2.3 Main reagents

High-fat feed (catalog number D12492) was purchased from Research Diets, Inc. (USA). AAV-shRNA-Lrg1 and AAV-shRNA-Control were purchased from Guangzhou Dongze Biotechnology Co., Ltd. (China). siLrg1 was purchased from Shanghai Jierui Biotechnology Co., Ltd. (China). Transfection reagent lipofectamine 2000 was purchased from Invitrogen (USA). DMEM/F12 medium and 0.25% trypsin were purchased from Gibco (USA). Fetal bovine serum was purchased from Pronase Life Science Technology Co., Ltd (China). p-AKT (Ser473) and AKT antibodies were purchased from Cell Signaling Technology (USA). Other antibodies were purchased from Proteintech (China). Triglyceride (TG) assay kit (catalog number A110-1-1), total cholesterol (TC) assay kit (catalog number A111-1-1), Alanine aminotransferase (ALT) assay kit (catalog number C009-2-1), and aspartate aminotransferase (AST) assay kit (catalog number C010-2-1) were purchased from Nanjing Jiancheng Bioengineering Research Institute (China).

### 2.4 Experimental methods

#### 2.4.1 RNA library preparation and sequencing analysis

Transcriptome sequencing was performed by Guangzhou Epigenomics Technology Co., Ltd. The sequencing samples were divided into four groups: CD, iCD, HFD, and iHFD, with three replicate samples in each group. Total RNA was extracted using TRIzol reagent following the manufacturer’s instructions. RNA purity, concentration, and integrity were assessed using a NanoDrop spectrophotometer (Thermo Scientific) and an Agilent Bioanalyzer 2100 (Agilent Technologies, Santa Clara, CA, US). Sequencing libraries were constructed using the Illumina platform with a standard protocol. Sequencing data were filtered using the fastp (https://github.com/OpenGene/fastp), and reference genome alignment was performed using the HISAT2 (v2.0.5).

#### 2.4.2 Differential analysis and enrichment analysis

Differential analysis was performed using the DESeq2 R package. Differentially expressed genes were selected based on |log2FC| > 1 and FDR < 0.05. These genes were then visualized using volcano plots and heat maps. Based on the “clusterProfiler” R package, KEGG, GO and GSEA enrichment analysis was carried out. The visual analysis is completed by the online platform of Micro-credit (https://www.bioinformatics.com.cn/) (13).

#### 2.4.3 Weighted gene co-expression network analysis and screening of core genes

We used WGCNA to identify co-expressed gene modules. Then, we studied their relationship with corresponding phenotypes to identify key genes that play a crucial role. WGCNA was performed using the online platform ImageGP (https://www.bic.ac.cn/BIC) (14). Based on the correlation coefficient (cor) and P-value, modules with higher correlation were identified. GO enrichment analysis was performed on the corresponding gene modules, with a focus on biological processes. To identify key genes involved in IF alleviating MAFLD, we intersected genes from the highest correlation module with DEGs of HFD vs CD and DEGs of iHFD vs HFD. The above genes were submitted to the String database (https://cn.string-db.org/) for protein-protein interaction (PPI) network analysis. The PPI network was visualized using Cytoscape software, and degree values were calculated. Genes were ranked based on degree values to identify core genes. Core genes were validated via RT-qPCR experiments.

#### 2.4.4 RT-qPCR

Total RNA was extracted from AML12 cells or mouse liver tissue with TRIzol reagent. After measuring the RNA concentration and purity, the RNA was reverse transcribed into cDNA using a reverse transcription kit. SYBR Green pre-mix was used to detect target genes on a real-time fluorescent quantitative PCR system. β-actin was used as the internal control, and the relative expression levels of target genes were calculated using the 2-ΔΔCt method. Primer sequences are listed in **Supplementary Table 1**.

#### 2.4.5 Western blotting

AML12 cells or mouse liver tissues were lysed using a buffer prepared with RIPA, protease inhibitor, and phosphatase inhibitor mixed at a ratio of 100:1:1. The protein concentration of the lysate was quantified by BCA protein assay kit. The protein supernatant was mixed with the 5× loading buffer and heated in a metal bath at 95–100°C for 10 minutes. Thirty micrograms of total protein were loaded for gel electrophoresis. After transferring the proteins to a PVDF membrane, the membrane was blocked at room temperature for 1 hour and then incubated overnight at 4°C with the corresponding primary antibody (1:1000). After incubation, the membrane was washed three times with TBST, incubated with the secondary antibody (1:10,000) for 1 hour at room temperature, and then washed again. The multifunctional molecular imaging system (Alliance Q9) was used for imaging, and the gray intensity of protein bands was measured by Image J software for quantitative analysis.

#### 2.4.6 HE staining of liver tissue

Mouse liver tissue was fixed in 4% paraformaldehyde solution. After dehydration, clearing, paraffin embedding and sectioning, the tissue was deparaffinized with xylene following standard protocols. The sections were stained with hematoxylin and 0.5% eosin, dehydrated, and mounted with neutral resin. The sections were observed under a microscope and photographed.

#### 2.4.7 Oil red O staining of liver tissue

Fresh liver specimens were snap-frozen. Then, 5–10 µm cryosections were prepared. After air-drying at room temperature, the sections were briefly rinsed with 70% ethanol and then immersed in freshly filtered Oil Red O working solution for 10–15 min. Following a gentle rinse with distilled water, nuclei were counterstained with Mayer’s hematoxylin for 3–5 min, after which excess stain was removed with running tap water to allow bluing. The sections were mounted with glycerol gelatin, and representative photomicrographs were acquired under a light microscope.

#### 2.4.8 Cell oil red O staining

After the specified treatment, the medium was aspirated from the 12-well plates. The cells were then gently rinsed three times with PBS. The cells were then fixed with 4% paraformaldehyde for 10–15min at room temperature and subsequently stained with freshly filtered Oil Red O working solution for 10–20min. Following three additional PBS washes, nuclei were counterstained with hematoxylin. Finally, an appropriate volume of PBS was added to the wells, and representative images were captured under a light microscope.

#### 2.4.9 Detection of serum TG, TC, AST, ALT, and liver TG and TC contents

The reagent kits were purchased from Nanjing Jiancheng Biotechnology Research Institute, and all indicators were measured according to the corresponding reagent kit instructions.

#### 2.4.10 Statistical analysis

The results are represented as mean ± SEM. GraphPad Prism 8.0 was used for statistical significance analysis and visualization. Two-tailed Student’s T-test was used for comparison between the two groups, and one-way ANOVA followed by Tukey’s multiple comparison was used for comparison between multiple groups. Statistical significance was defined as *p* < 0.05.

## 3 Results

### 3.1 IF improves glucose metabolism abnormalities and hepatic lipid deposition in MAFLD mice

**Figure 1:**
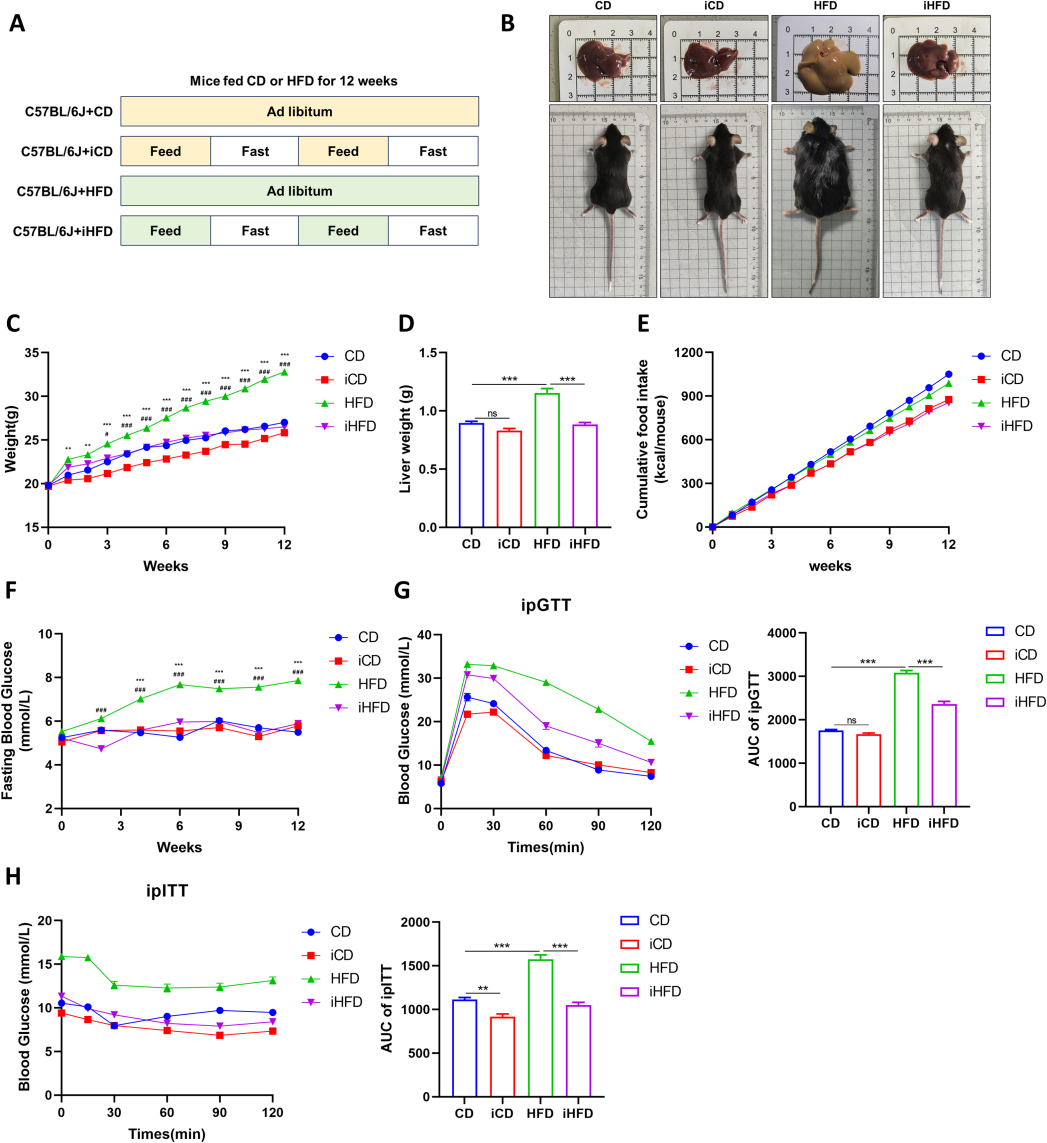
After IF, mice exhibited reduced fasting blood glucose levels, improved glucose tolerance, and reduced insulin resistance. **(A)** Mouse intervention methods. **(B)** Representative macroscopic images of mice and livers. **(C)** Mouse weight gain. **(D)** Liver weight. **(E)** Cumulative food intake. **(F)** Fasting blood glucose. **(G)** Intraperitoneal glucose tolerance test (ipGTT) curves and area under the curve (AUC). **(H)** Intraperitoneal insulin tolerance test (ipITT) curves and AUC. CD vs HFD, *p < 0.05, **p < 0.01, ***p < 0.001. HFD vs iHFD, #*p* < 0.05, ##*p* < 0.01, ###*p* < 0.001.

Compared with the CD group, HFD-fed mice exhibited higher body weight, liver weight, and fasting blood glucose (**Figures 1B–D, F**). ipGTT and ipITT analyses revealed markedly impaired glucose tolerance and aggravated insulin resistance in the HFD group relative to the CD group (**Figures 1G, H**). In contrast, compared with the HFD group, the iHFD group exhibited reduced body and liver weights, lower fasting glucose levels, and markedly improved glucose tolerance and insulin sensitivity (**Figures 1B–D, F–H**).

**Figure 2:**
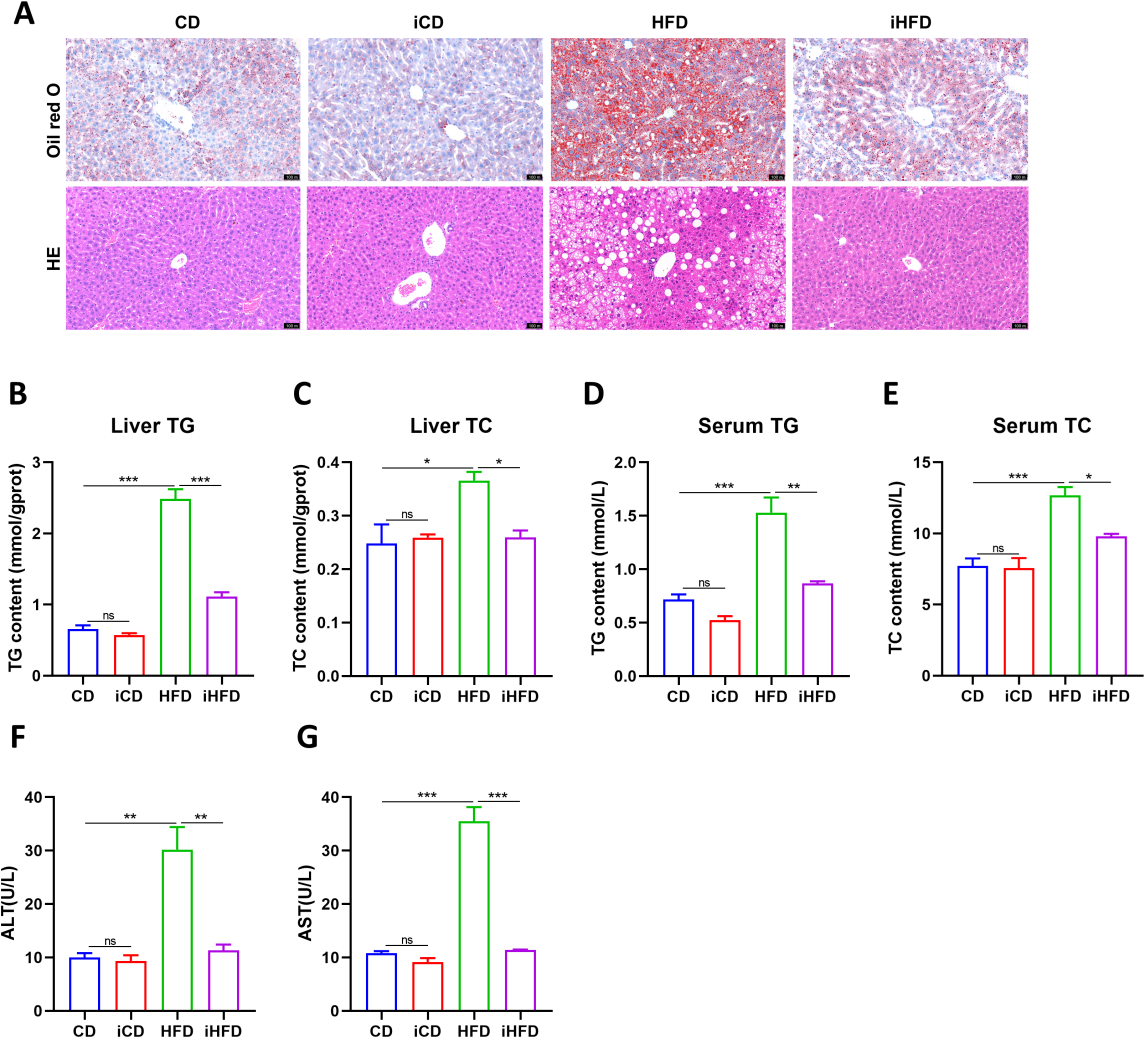
IF improved lipid deposition in mouse livers and reduced liver damage. **(A)** Oil red O (top) and HE staining (bottom) of mouse livers (20×). **(B, C)** TG and TC content in mouse liver tissue. **(D, E)** Serum TG and TC content. **(F, G)** Serum ALT and AST content. *p<0.05, **p<0.01, ***p<0.001.

HE and Oil Red O staining showed that hepatic lipid accumulation was markedly increased in HFD-fed mice compared to CD controls (**Figure 2A**). Consistently, hepatic triglyceride and total cholesterol contents increased significantly in the HFD group (**Figures 2B, C**), and serum triglyceride and total cholesterol levels also rose correspondingly (**Figures 2D, E**). Serum activities of the liver injury markers AST and ALT were also higher in HFD mice (**Figures 2F, G**). Relative to HFD mice, IF intervention (iHFD) markedly attenuated hepatic lipid deposition and alleviated liver injury (**Figures 2A–G**). Collectively, these data indicate that IF effectively ameliorates high-fat diet–induced hepatic steatosis in MAFLD.

### 3.2 Identification of DEGs

**Figure 3:**
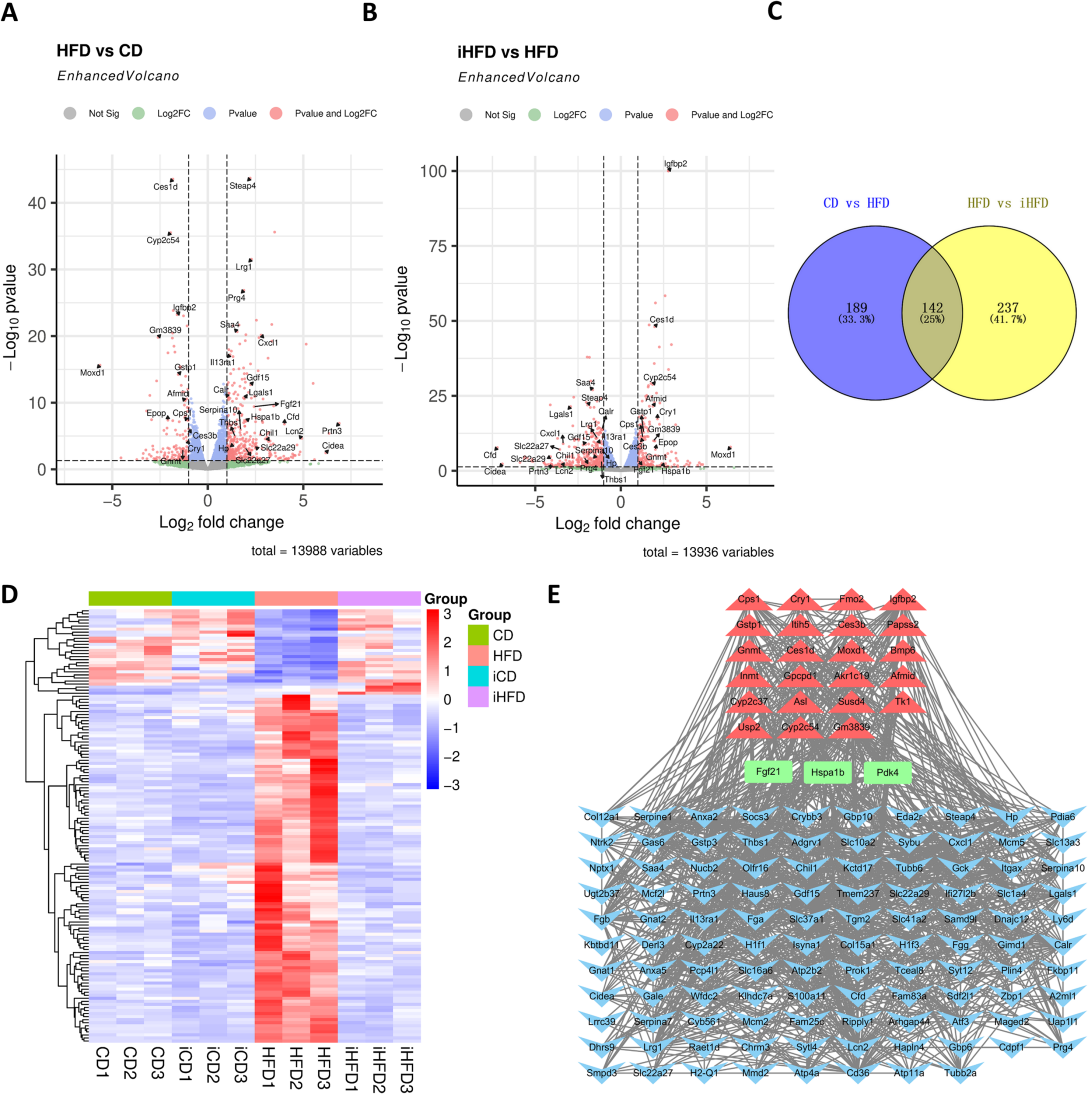
DEGs between groups of mice. **(A)** DEGs between the HFD and CD groups. **(B)** DEGs between the iHFD and HFD groups. **(C)** Venn diagram of DEGs between HFD vs CD and HFD vs CD. **(D)** Heat map of DEGs obtained in Figures **(C, E)**. Interaction network diagram of the DEGs obtained from Figure **(C)** Triangles represent DEGs upregulated in iHFD vs HFD (downregulated in HFD vs CD), V-shapes represent DEGs downregulated in iHFD vs HFD (upregulated in HFD vs CD), and rectangles represent DEGs upregulated in both HFD vs CD and HFD vs CD.

DEGs were identified using the criteria | log2FC | > 1 and FDR < 0.05. Comparison of HFD versus CD livers yielded 331 DEGs, including 79 up-regulated and 252 down-regulated transcripts (**Figure 3A**). In the iHFD versus HFD comparison, 379 DEGs were detected, with 139 up-regulated and 240 down-regulated (**Figure 3B**). Intersection analysis of these two DEG sets revealed 142 common genes (**Figure 3C**), which were then visualized using a clustered heat map. The expression patterns in HFD vs CD were almost completely reversed in iHFD vs HFD (**Figure 3D**). These reversed genes include those closely related to lipid metabolism, such as Igfbp2, Ces1d, Cyp2c54, Ces3b, Fgf21, Cyp2c37 and Lrg1, and genes closely related to inflammation, such as Il13ra1, Lgals1, Cxcl1, Prtn3, and Hp (**Figure 3E**). These findings indicate that IF may alleviate MAFLD by modulating gene networks related to lipid metabolism and inflammation.

### 3.3 KEGG and GO enrichment analysis

**Figure 4:**
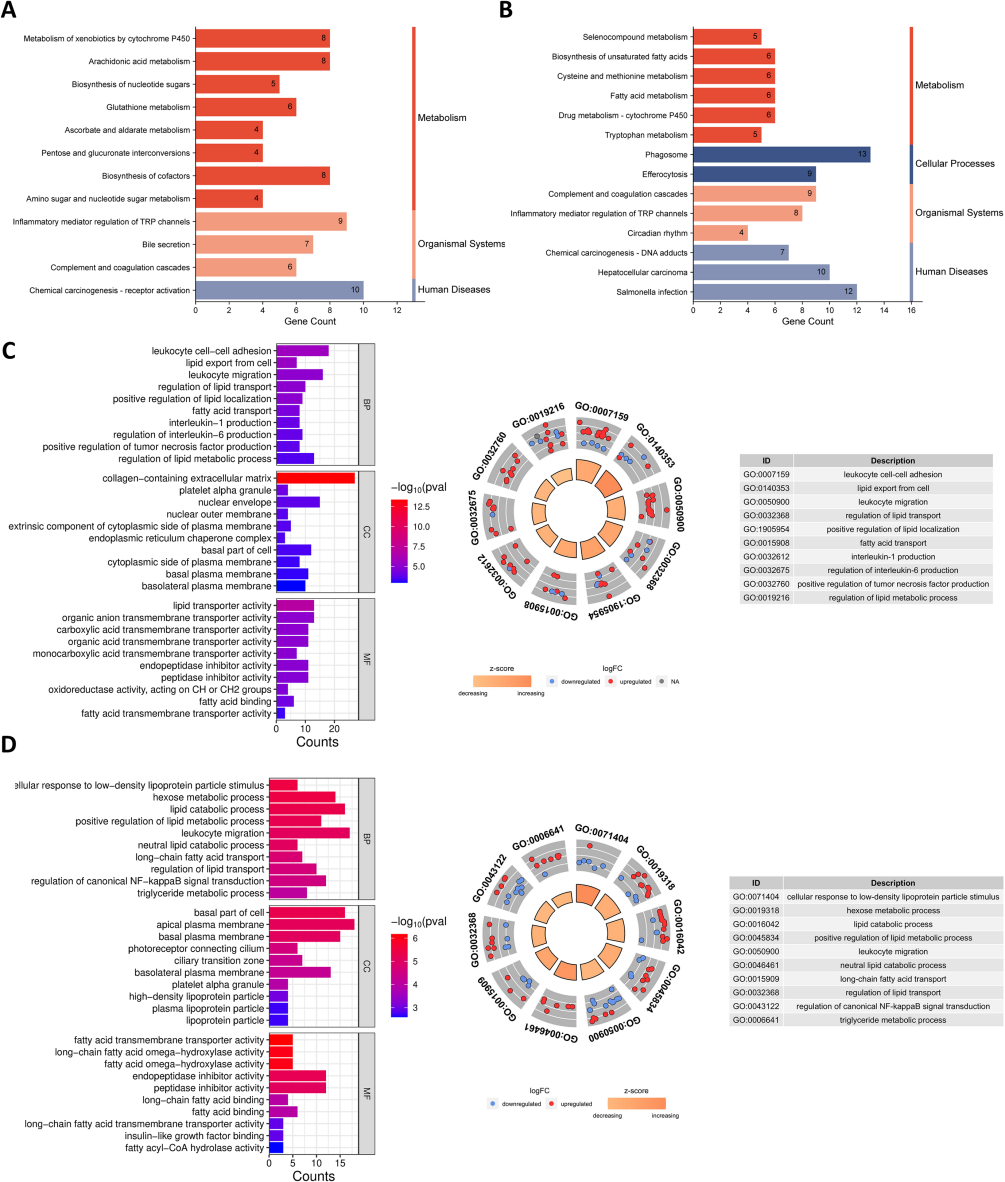
KEGG and GO enrichment analysis of DEGs. **(A)** KEGG enrichment analysis of DEGs between HFD and CD. **(B)** KEGG enrichment analysis of DEGs between iHFD and HFD. **(C)** GO enrichment analysis of DEGs between HFD and CD and the pie chart of BP enrichment results. **(D)** GO enrichment analysis of DEGs between iHFD and HFD and the pie chart of BP enrichment results.

KEGG enrichment analysis indicated that DEGs were significantly enriched in Biosynthesis of unsaturated fatty acids, Fatty acid metabolism, Inflammatory mediator regulation of TRP channels, and Arachidonic acid metabolism. Most of these pathways belonged to the Metabolism category in the KEGG Pathway classification (**Figures 4A, B**). GO enrichment was categorized as biological process (BP), cellular component (CC), and molecular function (MF) (**Figures 4C, D**). BP enrichment includes entries related to lipid metabolic processes and inflammatory responses, such as lipid export from cell, fatty acid transport, lipid catabolic process, interleukin-1 production, regulation of interleukin-6 production, and regulation of canonical NF-kappaB signal transduction. CC enrichment includes terms such as collagen-containing extracellular matrix, plasma lipoprotein particle, and lipoprotein particle. MF enrichment includes terms such as carboxylic acid transmembrane transporter activity, fatty acid binding, fatty acid transmembrane transporter activity, and long-chain fatty acid binding.

### 3.4 GSEA enrichment analysis

**Figure 5:**
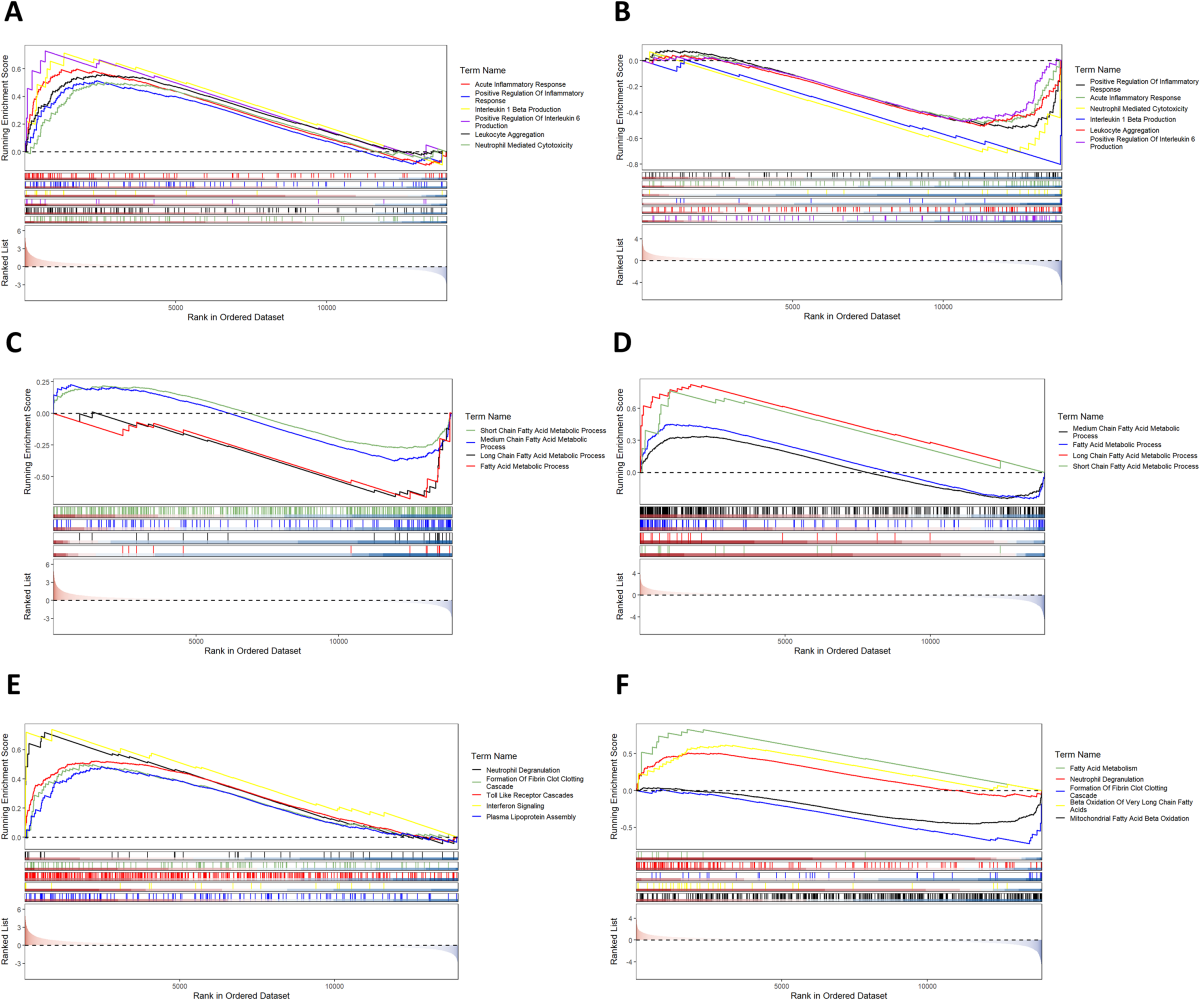
GSEA enrichment analysis results. **(A)** Upregulated inflammation-related pathways in the GOBP database enrichment analysis between the HFD and CD group. **(B)** Downregulated inflammation-related pathways in the GOBP database enrichment analysis between the iHFD and HFD group. **(C)** Downregulated fatty acid metabolism pathways in the HFD vs CD group in the GOBP database enrichment analysis. **(D)** Upregulated fatty acid metabolism pathways in the iHFD vs HFD group in the GOBP database enrichment analysis. **(E)** Inflammation and fatty acid metabolism-related pathways in the HFD vs CD group in the Reactome database enrichment analysis. **(F)** Inflammation and fatty acid metabolism-related pathways in the iHFD vs HFD group in the Reactome database enrichment analysis.

GSEA retains more biological information through genome-wide sequence analysis and identifies biological pathways with weak multi-gene synergistic effects. According to the enrichment results from the GOBP database, compared with the CD group, the HFD group showed significant up-regulation of gene sets associated with inflammatory response, whereas gene sets linked to fatty acid metabolic process were down-regulated (**Figures 5A, C**). Compared with the HFD group, IF intervention (iHFD group) reversed these signatures: inflammatory response pathways were down-regulated, while fatty acid metabolism pathways were up-regulated (**Figures 5B, D**). Parallel GSEA using the Reactome database confirmed these findings, revealing concordant enrichment patterns for both inflammation-related and fatty acid metabolism pathways (**Figures 5E, F**). This suggests that IF may alleviate MAFLD by reducing inflammatory responses and promoting fatty acid metabolism.

### 3.5 Construction of WGCNA

**Figure 6:**
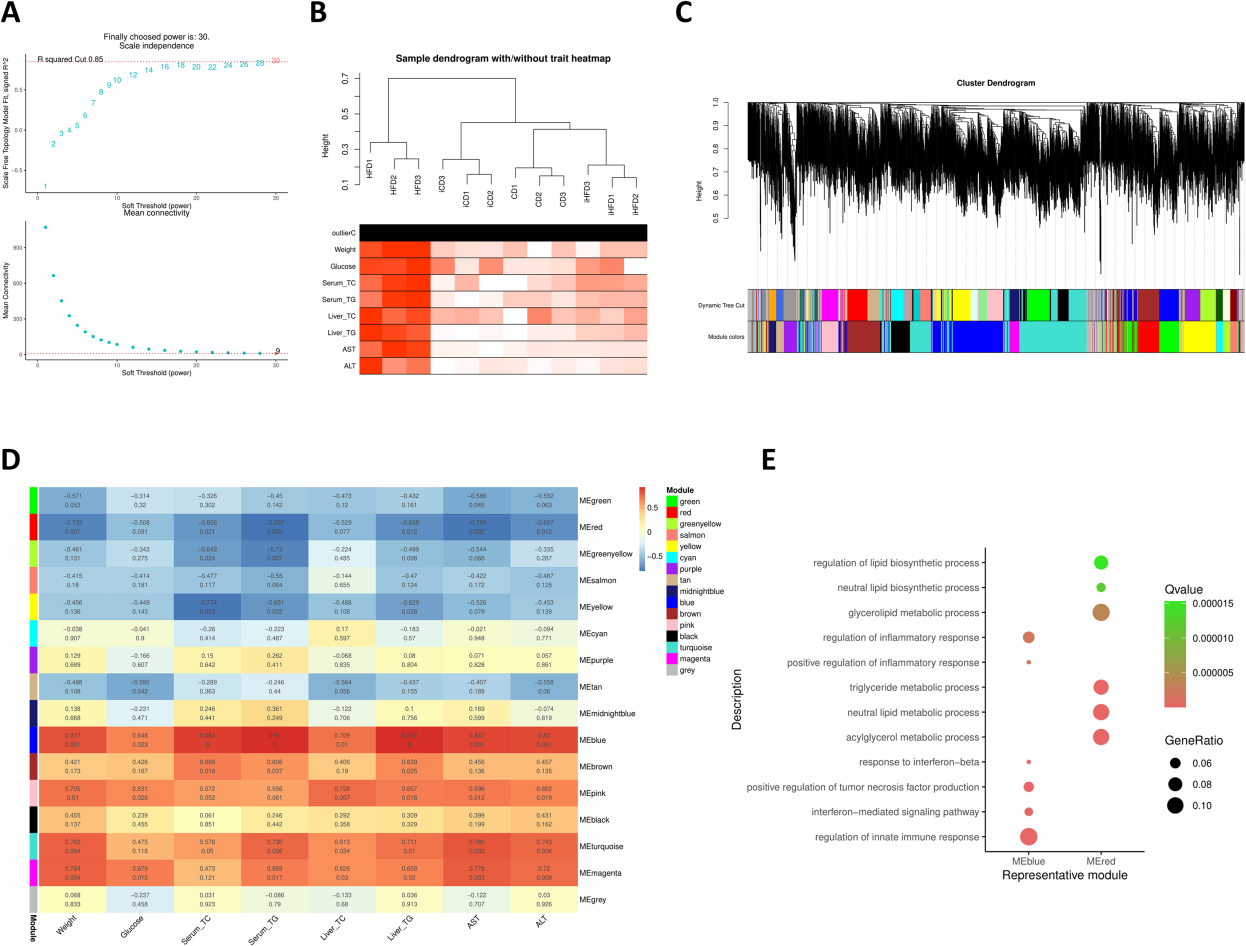
Weighted gene co-expression network. **(A)** The upper panel shows the scale-free topological model fit (R2 value) under different soft threshold powers, and the lower panel shows the average connectivity under different soft threshold powers. **(B)** Hierarchical clustering showing sample correlations. **(C)** Gene clustering dendrogram and module partitioning. Each module is represented by a different color. **(D)** Correlation analysis between modules and phenotypes. Each row represents a module. Each column represents a phenotype (trait attribute). Blue indicates negative correlation, and red indicates positive correlation. **(E)** Enrichment analysis results for genes in blue and red modules.

To uncover relationships between gene modules and MAFLD-related phenotypes and to identify putative regulatory hubs, we constructed a weighted gene co-expression network using WGCNA. The soft threshold was set to 30, and the corresponding R2 value was 0.85, generating a network with high average connectivity (**Figure 6A**). Clustering dendrograms of sample-relatedness and gene-relatedness were generated, and 16 gene modules were detected (**Figures 6B, C**). Based on correlation coefficient and P value, the red and blue modules exhibited the strongest associations (**Figure 6D**). Functional interrogation of these two modules by GO enrichment analysis, with emphasis on BP terms, revealed divergent biological roles. The blue module was enriched for inflammation- and immune-related pathways, such as interferon-mediated signaling pathway, whereas the red module was enriched for lipid-metabolic processes, such as triglyceride metabolic process (**Figure 6E**). These enrichment results indicate that the two modules play key roles in inflammation, immunity, and lipid metabolism—critical pathophysiological processes underlying MAFLD progression.

### 3.6 Screening and identification of key genes

**Figure 7:**
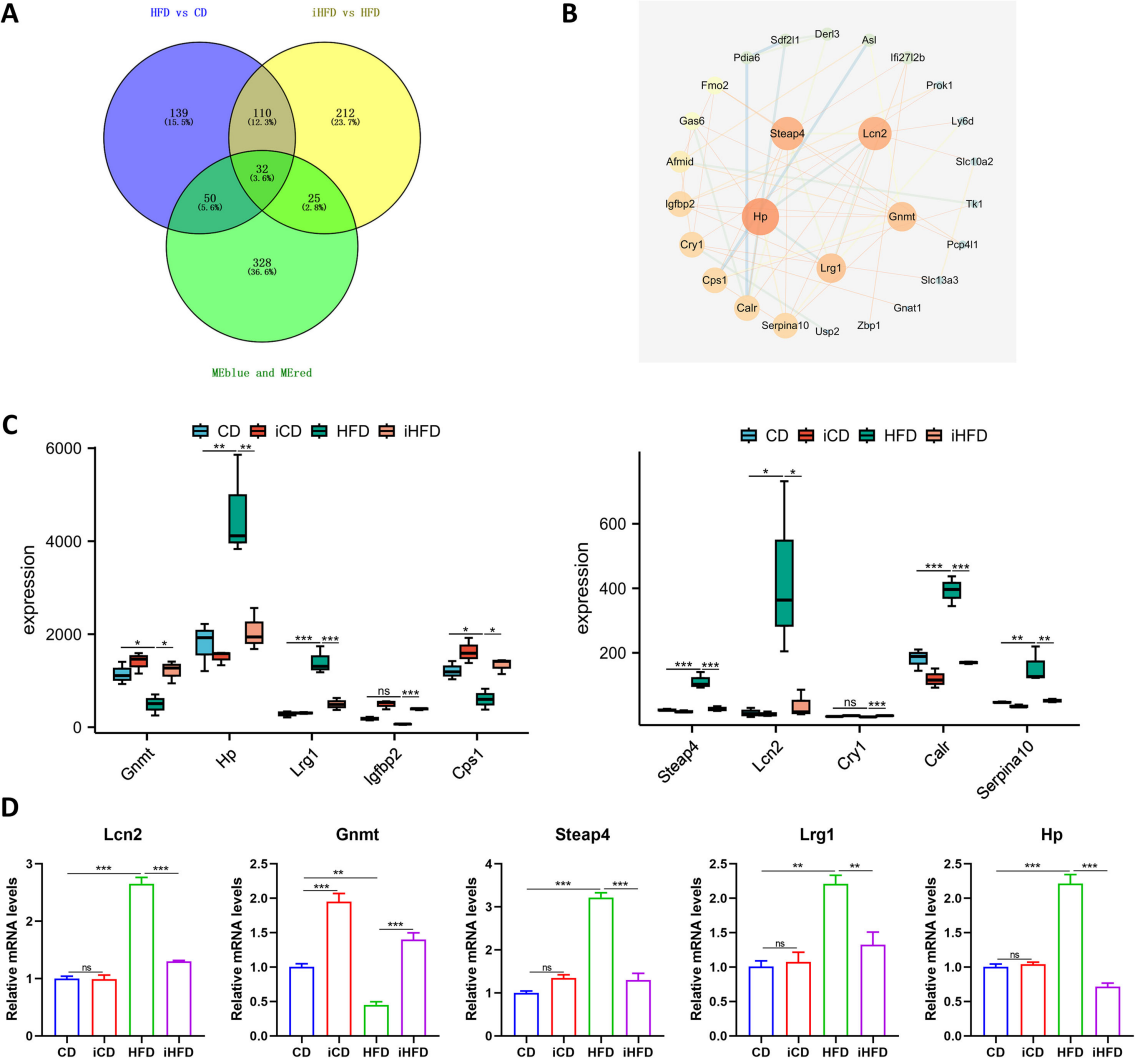
Screening and identification of key genes. **(A)** Venn diagram showing the overlap between genes of red and blue modules, DEGs of HFD vs CD, DEGs of iHFD vs HFD. **(B)** PPI network analysis of the genes obtained in Figure **(A) (C)** Box plot showing the expression levels of the top 10 key genes. **(D)** RT-qPCR experiment to validate the mRNA expression levels of the top 5 key genes. *p < 0.05, **p < 0.01, ***p < 0.001.

To identify the key genes through which IF alleviates MAFLD, we intersected the genes of red and blue modules, DEGs of HFD vs CD and DEGs of iHFD vs HFD, yielding 32 common genes (**Figure 7A**). A PPI network was subsequently constructed for these genes using the STRING database (**Figure 7B**), and the top ten key genes were selected on the basis of degree centrality. Box plots show the expression levels of these genes across the four experimental groups (**Figure 7C**). We extracted RNA from mouse liver tissue and analyzed the first five genes using qPCR. Compared with the CD group, lipocalin 2 (Lcn2), STEAP family member 4 (Steap4), Lrg1, and haptoglobin (Hp) were upregulated, while glycine N-methyltransferase (Gnmt) was downregulated in the HFD group. However, compared with the HFD group, these expression trends were reversed in the iHFD group (**Figure 7D**).

### 3.7 Hepatic Lrg1 knockdown alleviates MAFLD and potentiates the therapeutic effects of IF

**Figure 8:**
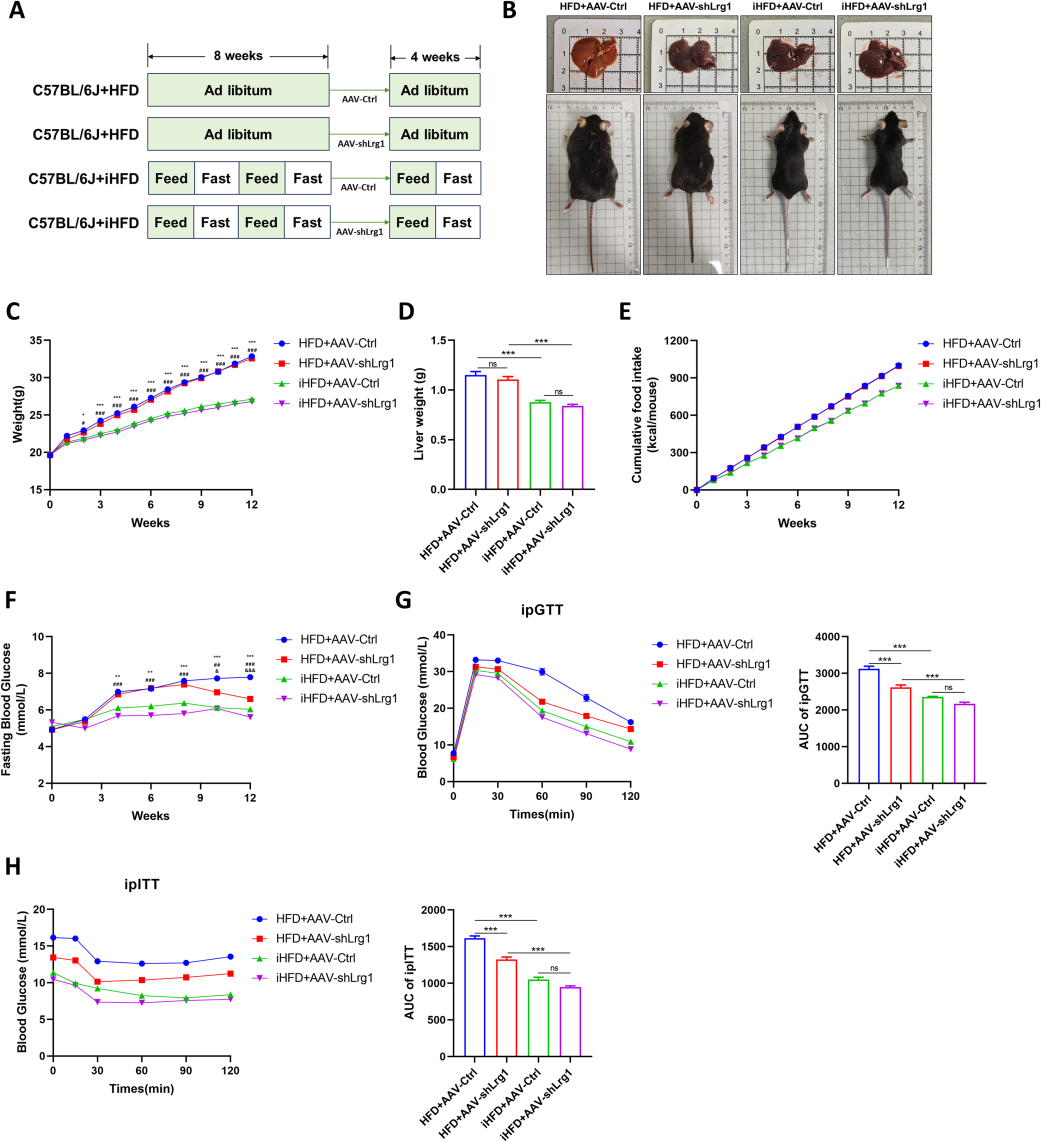
After liver-specific knockdown of Lrg1, mice exhibited reduced fasting blood glucose levels, improved glucose tolerance, and enhanced insulin sensitivity. **(A)** Mouse intervention method. **(B)** Representative macroscopic images of mice and livers. **(C)** Mouse weight gain. **(D)** Liver weight. **(E)** Cumulative food intake. **(F)** Fasting blood glucose. **(G)** ipGTT curves and AUC. **(H)** ipITT curves and AUC. HFD+AAV-Ctrl vs iHFD+AAV-Ctrl, *p<0.05, **p<0.01, ***p<0.001. HFD+AAV-shLrg1 vs iHFD+AAV-shLrg1, #*p*<0.05, ##*p*<0.01, ###*p*<0.001. iHFD+AAV-Ctrl vs iHFD+AAV-shLrg1, &*p* < 0.05, &&*p* < 0.01, &&&*p* < 0.001.

**Figure 9:**
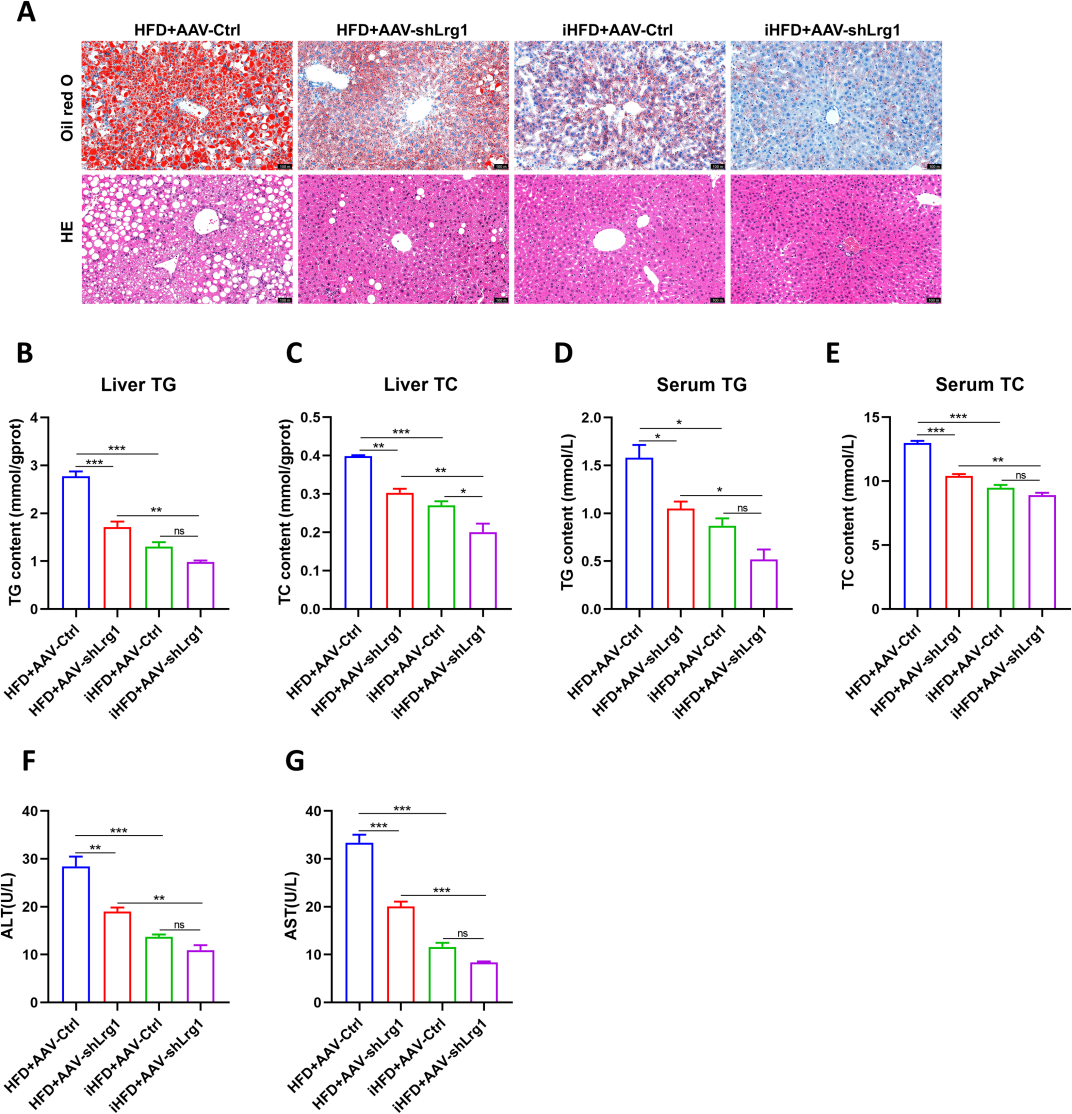
Liver lipid deposition in MAFLD mice was improved and liver injury was reduced after liver knockdown of Lrg1. **(A)** Oil red O (top) and HE staining (bottom) of mouse liver tissue (20×). **(B, C)** TG and TC levels in mouse liver tissue. **(D, E)** Serum TG and TC levels. **(F, G)** Serum ALT and AST levels. *p < 0.05, **p < 0.01, ***p < 0.001.

Based on the fundamental functions of key genes and differences in their transcriptomic expression, we selected Lrg1 for further investigation. Lrg1 was recently identified as an adipokine involved in lipid metabolism and energy homeostasis. The HFD+AAV-shLrg1 group showed no significant change in body and liver weight compared to the HFD+AAV-Ctrl group (**Figures 8C, D**) but had markedly reduced fasting blood glucose (**Figure 8F**). ipGTT and ipITT revealed improved glucose tolerance and attenuated insulin resistance in the HFD+AAV-shLrg1 group relative to HFD+AAV-Ctrl controls (**Figures 8G, H**). HE and Oil Red O staining showed a pronounced reduction in hepatic lipid droplet accumulation in the HFD+AAV-shLrg1 group (**Figure 9A**). This was accompanied by significant decreases in hepatic triglyceride and total cholesterol levels (**Figures 9B, C**), as well as corresponding reductions in serum triglyceride and total cholesterol levels (**Figures 9D, E**). Additionally, serum AST and ALT levels were lower in the knockdown group (**Figures 9F, G**).

Compared to the HFD+AAV-Ctrl group, the iHFD+AAV-Ctrl group showed lower fasting blood glucose, less hepatic lipid deposition, and reduced liver damage. Compared with the iHFD+AAV-Ctrl group, the iHFD+AAV-shLrg1 group showed further improvement in hepatic lipid deposition (**Figures 8**, **9**). In summary, Lrg1 knockdown in the liver significantly slows MAFLD progression and enhances the alleviating effect of IF.

### 3.8 Hepatic Lrg1 knockdown suppresses lipogenic gene expression and alleviates hepatocellular lipid infiltration

**Figure 10:**
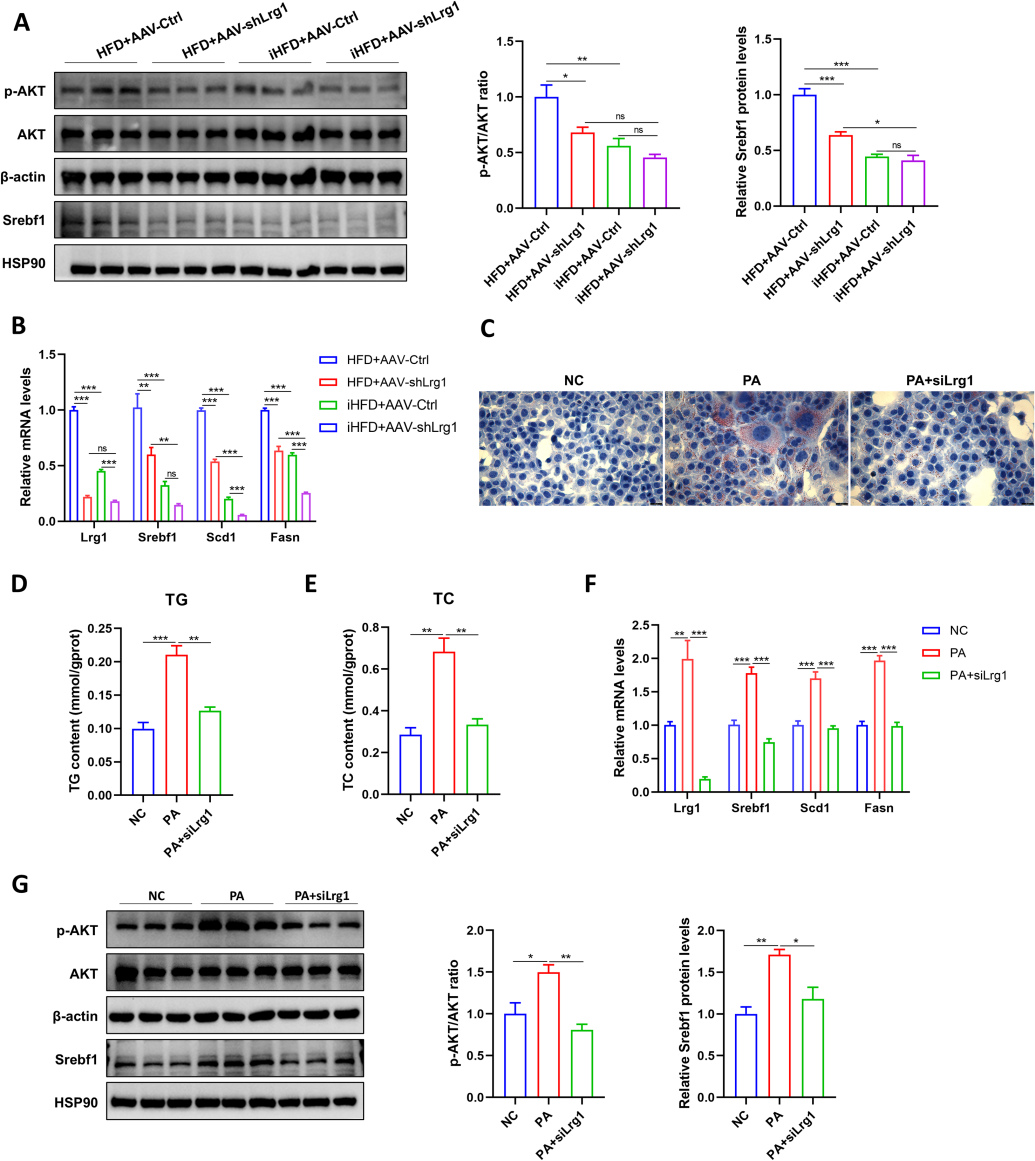
Knockdown of Lrg1 inhibits the expression of lipid synthesis genes and alleviates hepatic lipid infiltration. **(A)** Effects of Lrg1 knockdown in the liver on the expression of p-AKT, AKT, and Srebf1 proteins. **(B)** Effects of Lrg1 knockdown in the liver on the expression of Srebf1, Scd1, and Fasn mRNA. **(C)** Oil red O staining to observe the effect of Lrg1 knockdown on PA-induced lipid deposition in AML12 cells (40×). **(D)** Effect of Lrg1 knockdown on TG and TC levels in AML12 cells. **(F)** Effect of Lrg1 knockdown on Srebf1, Scd1, and Fasn mRNA expression in AML12 cells. **(G)** Serum ALT and AST levels. **(G)** Effects of Lrg1 knockdown in AML12 cells on p-AKT, AKT, and Srebf1 protein expression. *p < 0.05, **p < 0.01, ***p < 0.001.

Loss of Lrg1 suppressed AKT phosphorylation and decreased sterol regulatory element binding transcription factor 1 (Srebf1) expression (**Figures 10A, B**). Similarly, the transcript levels of downstream lipogenic genes, including stearoyl-Coenzyme A desaturase 1 (Scd1) and fatty acid synthase (Fasn), were also reduced (**Figure 10B**). *In vitro*, cells with Lrg1 knockdown showed attenuated lipid accumulation compared to the PA group (**Figure 10C**), and lower intracellular triglyceride and total cholesterol levels (**Figures 10D, E**). Western blotting and qPCR analyses further revealed a reduced p-AKT/AKT ratio and diminished expression of Srebf1, Scd1, and Fasn (**Figures 10F, G**). These findings suggest that Lrg1 deficiency may inhibit Srebf1 expression via the PI3K-AKT pathway, which in turn reduces the expression of downstream genes Scd1 and Fasn, ultimately decreasing lipid accumulation in hepatocytes.

## 4 Discussion

This study aims to explore the potential and mechanisms of IF in improving MAFLD. Through transcriptomics analysis and *in vitro* and *in vivo* experiments, the effect of IF on MAFLD was systematically evaluated, and its regulation on lipid metabolism and inflammatory response was emphatically analyzed. The results showed that IF significantly improved the metabolic abnormalities and reduced liver lipid deposition of MAFLD mice. Key DEGs related to these processes were identified, and experiments confirmed that Lrg1, one of the key genes, plays a regulatory role in the progression of MAFLD.

The results of this study showed that IF significantly improves MAFLD, particularly by ameliorating metabolic abnormalities and reducing liver lipid deposition. Compared with the HFD group, mice in the iHFD group showed lower fasting blood glucose, AST, and ALT levels, improved glucose tolerance, reduced insulin resistance, and significantly decreased hepatic lipid infiltration. These results are consistent with previously reported studies, indicating that IF alleviates MAFLD by improving metabolic syndrome (15, 16).

KEGG and GO enrichment analysis showed that DEGs were significantly enriched in fatty acid metabolism and inflammation- related pathways, which emphasized the potential mechanism of IF in improving MAFLD. GSEA analysis results showed that, compared with the CD group, the inflammatory response pathway was significantly upregulated in the HFD group, while the fatty acid metabolism pathway was significantly downregulated. Compared with the HFD group, the inflammatory response pathway was significantly downregulated in the iHFD group, while the fatty acid metabolism pathway was significantly upregulated. The role of IF in metabolic dysfunction-associated fatty liver disease has been widely reported, particularly in terms of regulating inflammation and lipid metabolism. IF can reduce the expression and concentration of various inflammatory markers, including C-reactive protein (CRP), tumor necrosis factor-α (TNF-α), and interleukin-6 (IL-6) (8, 11, 17). Additionally, it can also promote fat β-oxidation and inhibit fat synthesis by regulating AMPK, PPAR and mTOR signals (8, 18, 19). RNA sequencing of the liver also revealed significant enrichment of fatty acid metabolism and inflammation-related pathways (19). In addition, the enrichment results also suggested the role of immune responses, oxidative stress, and other pathways in the influence of IF on MAFLD.

We identified 331 DEGs between HFD and CD group and obtained 379 DEGs in iHFD and HFD group. Among these, 142 DEGs were common to both comparisons. Interestingly, the common DEGs showed opposite expression patterns between the two groups, suggesting that IF may reverse the progression of MAFLD (20, 21). WGCNA revealed correlations between gene modules and phenotypes. Specifically, the blue module was associated with inflammation and immune response, while the red module was related to lipid metabolism. By integrating the differential gene expression data and module-related genes, we identified the differential genes with strong correlation according to PPI, among which Lcn2, Gnmt, Steap4, Hp and Lrg1 have the highest correlation. It is reported that the expression of Lcn2 is significantly upregulated in patients with non-alcoholic steatohepatitis. In animal models, increased hepatic Lcn2 correlates closely with multiple interleukins and Toll-like receptors. Lcn2 may mediate MAFLD progression through inflammatory responses (22). In addition, Lcn2 regulates the immune environment of the liver by influencing cytokine release, and it intensifies the inflammatory reaction of the liver (23). GNMT knockout (KO) mice exhibit significantly elevated SAM levels, leading to hepatic lipid accumulation and impaired oxidative metabolism. Methionine-restricted diets prevent increased SAM levels, improved gluconeogenesis and oxidative phosphorylation, and mitigated MAFLD progression. The hepatic metabolic dysfunction caused by GNMT deficiency partially depends on altered SAM levels and affects downstream gene expression by influencing the availability of methylation donors (24). STEAP4 is a redox enzyme capable of oxidizing ferrous iron (Fe^2+^) to ferric iron (Fe^3+^) and participates in iron uptake. Inflammation triggered by bacterial lipopolysaccharide (LPS) disrupts iron homeostasis in adipocytes by upregulating STEAP4, leading to abnormal intracellular iron accumulation (25). Steap4 further enhances NF-κB activity by promoting iron accumulation and oxidative stress, leading to increased inflammation (26). Hp expression is elevated in mice with NASH induced by a high-fat diet (27). In addition, clinical studies show that Hp concentration is positively correlated with serum IL-6 and 8-isoprostane concentrations (28). Hp amplifies inflammatory reaction and signal transduction through Mincle, and promotes the progress of inflammation (29). The results reported above indicate that these key genes play an role in the occurrence and development of MAFLD. This suggests that intermittent fasting may regulate the progression of MAFLD through these genes.

However, although the aforementioned four molecules are associated with MAFLD, there is a lack of strong direct evidence linking them to the core pathology of MAFLD. They may participate in MAFLD progression as secondary effectors or by engaging in auxiliary regulatory pathways. In transcriptomic data, Lrg1 exhibited significant expression differences and high statistical significance across groups and was closely associated with hepatic lipid metabolism and inflammation. Therefore, we have identified Lrg1 as a key gene for further investigation. We found that knocking down its expression can alleviate MAFLD in experimental models. Lrg1 is a leucine-rich glycoprotein involved in regulating lipid and carbohydrate metabolism, inflammatory responses, and fibrotic processes. Its expression is upregulated in various diseases, such as specific types of cancer, diabetes, and cardiovascular diseases, and these levels are closely associated with disease prognosis (30, 31). It has been pointed out that the increase of serum Lrg1 induced by HFD can aggravate hepatic lipomatosis by inhibiting fatty acid β oxidation and promoting *de novo* lipogenesis in mice. The binding of Lrg1 to hepatic cells induces hyperglycemia by inhibiting insulin signaling and promoting gluconeogenesis (32). Mice lacking Lrg1 show significant relief from diet-induced hepatic steatosis, obesity, and insulin resistance (32). Our study found that silencing Lrg1 in the liver using a genetic knockdown model improved HFD-induced hepatic steatosis, hyperglycemia, and insulin resistance. This intervention also further enhanced the beneficial effects of IF on MAFLD. Additionally, we demonstrated *in vitro* that reducing Lrg1 expression in hepatocytes alleviated PA-induced cellular steatosis. Furthermore, we found that knocking down Lrg1 inhibited the phosphorylation of the PI3K-AKT pathway and the expression of lipogenic genes. The above results indicate that, in addition to Lrg1 in the peripheral circulation, hepatocyte-derived Lrg1 also plays an important regulatory role in hepatic steatosis. Lrg1 can serve as a potential intervention target for MAFLD and, as a protein widely present in peripheral circulation, also function as a marker for its screening and diagnosis.

The pivotal comorbidity of MAFLD is cardiovascular disease (CV disease). Lrg1 and IF both influence atherosclerosis, the main driver of CV diseases. First, Lrg1 acts as a regulator of the TGF-β signaling pathway, selectively activating specific pathways by altering the downstream molecules of TGF-β signaling. Under normal physiological conditions, TGF-β1 primarily inhibits cell proliferation and maintains vascular homeostasis through the ALK5/Smad2/3 pathway. However, when Lrg1 levels are elevated, it forms a complex with TGF-β1, recruits the co-receptor Endoglin, and promotes activation of the ALK1/Smad1/5/8 pathway. This “pathway switching” redirects TGF-β signaling toward pro-angiogenic and pro-fibrotic pathways (33, 34). MAFLD itself is accompanied by hepatic inflammation. Lrg1 released from the liver enters the circulation, where it further amplifies TGF-β signaling in the vascular wall, promoting structural alterations and inflammatory infiltration (33, 35). Second, elevated Lrg1 promotes an inflammatory environment (36). By altering the cytoskeleton of endothelial cells and upregulating the expression of adhesion molecules such as VCAM-1 and ICAM-1, Lrg1 facilitates the infiltration of monocytes and macrophages into the vascular wall, making them more “sticky” to L-selectin and other leukocytes (37). Third, Lrg1 activates the Smad1/5/8 pathway and the Wnt/β-catenin pathway in vascular smooth muscle cells (VSMCs), promoting cell proliferation, migration, and calcification. These activated VSMCs migrate into the intimal layer, forming fibrous caps that lead to carotid intima-media thickness (cIMT) (33, 38). In patients with obesity-related MAFLD, serum eotaxin levels and the severity of hepatic steatosis assessed by ultrasound predict carotid intima-media thickness. This finding reveals an association between MAFLD and atherosclerosis (39). Therefore, elevated Lrg1 levels resulting from hepatic steatosis disrupt the barrier function of the vascular endothelium by remodeling the TGF-β signaling pathway, thereby promoting abnormal proliferation and calcification of vascular smooth muscle cells. This process ultimately manifests as cIMT and the formation of atherosclerosis. On the other hand, IF helps reduce early atherosclerosis. IF promotes the body’s transition from glucose metabolism to fatty acid oxidation and ketone body production. Simultaneously, IF suppresses cholesterol synthase activity in the liver. These effects reduce serum levels of TC and LDL-C. The reduction in lipid levels directly decreases lipid deposition beneath the vascular endothelium, mitigates macrophage phagocytosis and foam cell formation triggered by oxidized low-density lipoprotein (Ox-LDL), thereby inhibiting the early formation of atherosclerotic plaques (40–42). Early atherosclerosis begins with immune cells (monocytes) adhering to the arterial wall. IF inhibits the expression of adhesion molecules (such as VCAM-1 and ICAM-1) on vascular endothelial cells by reducing serum levels of inflammatory factors like CCL2/MCP-1, which directly reduces the adhesion capacity of circulating monocytes, blocking their migration into the vascular wall. This fundamentally enhances the arterial wall’s resistance to plaque formation (40, 42). Lrg1 stimulates pro-inflammatory M1-like polarization of macrophages by activating the extracellular signal-regulated kinase 1/2 (ERK1/2) and c-Jun N-terminal kinase (JNK) pathways. In animal models, knockout of the Lrg1 gene or use of anti-Lrg1 neutralizing antibodies delays atherosclerotic progression (43). Furthermore, in mice fed a high-fat diet, Lrg1 knockout protects mice from diet-induced pancreatic hyperplasia and β-cell mass loss. Neutralizing Lrg1 activity prevents diet-induced hyperinsulinemia and preserves glucose tolerance (44). Our findings demonstrate that IF reduces Lrg1 expression in the livers of MAFLD mice. Knocking down hepatic Lrg1 in MAFLD mice suppresses the expression of hepatic lipid synthesis genes, significantly lowering both hepatic and serum lipid levels. These results suggest that Lrg1 participates in the regulation of metabolism and inflammation during intermittent fasting, alleviating MAFLD while also potentially slowing the progression of early atherosclerosis.

This study is limited by a relatively small sample size and lack of clinical data verification, which may reduce the generalizability of the results. In addition, verification of key genes is limited; except for Lrg1, functional experiments have not been conducted for other key genes. Therefore, future research should increase sample size and validate findings using clinical data as well as *in vitro* and *in vivo* experiments to enhance result reliability. Furthermore, the specific molecular mechanisms by which Lrg1 improves MAFLD and its mechanisms in IF require further investigation. The effects of IF on atherosclerosis in MAFLD through Lrg1 also requires further exploration.

## 5 Conclusions

This study used transcriptomics to show that IF improves MAFLD by regulating lipid metabolism and inflammatory responses. This provides a macro-level perspective on the mechanism of action of intermittent fasting. Additionally, several key genes involved in the regulation of MAFLD by intermittent fasting were identified, demonstrating their potential value for the diagnosis and intervention of MAFLD. Furthermore, the study found that knocking down the key gene Lrg1 could slow the progression of MAFLD, which lays a foundation for its basic research and clinical application.

## Data Availability

The sequencing data presented in this study are deposited in the National Center for Biotechnology Information (NCBI) Sequence Read Archive (SRA), with the accession number PRJNA1420219.

## Supplementary material

The supplementary information is in PDF documents named “Supplementary Figure” and “Supplementary Table”.
