# Supplementary material for: Intermittent fasting ameliorates MAFLD by downregulating Lrg1: insights from bulk RNA sequencing and functional verification
Source: Front Endocrinol (Lausanne). 2026 Feb 16;17:1754251. doi: 10.3389/fendo.2026.1754251 (PMC12950749; doi:10.3389/fendo.2026.1754251)
Supplement: Supplementary file 1 [file DataSheet1.pdf]

Supplementary Table 1. List of primer sequences.

Gene	Primers	Sequence
β-actin	Forward	ACTGCCGCATCCTCTTCCTC
	Reverse	AACCGCTCGTTGCCAATAGTG
Lrg1	Forward	GGGTACAACCTGCTGGAGTC
	Reverse	TATCCTCCAGCCTCTGCAGT
Lcn2	Forward	GGACTACAACCAGTTCGCCA
	Reverse	CTTGAGGCCCAGAGACTTGG
Gnmt	Forward	CCCACATGGTAACCCTGGAC
	Reverse	AGCCGGAACTTACTGAAGCC
Steap4	Forward	GCTCTGGGATTTGCCTTCCT
	Reverse	AGGGCCTGAGTAATGGTTGC
Hp	Forward	GGCTATGTGGAGCACTTGGT
	Reverse	TCTCCAGCGACTGTGTTCAC
Srebf1	Forward	TGACCCGGCTATTCCGTGA
	Reverse	CTGGGCTGAGCAATACAGTTC
Scd1	Forward	AGCCTGTTCGTTAGCACCTTCTTG
	Reverse	GCACCCAGGGAAACCAGGATATTC
Fasn	Forward	TGCCCGAGTCAGAGAACCTACAG
	Reverse	TCCATAGAGCCCAGCCTTCCATC

Supplementary Table 2. List of abbreviations.

Abbreviations	Full names
NAFLD	nonalcoholic fatty liver disease
IF	intermittent fasting
CD	normal ad libitum diet
HFD	high-fat ad libitum diet
GO	Gene Ontology
KEGG	Kyoto Encyclopedia of Genes and Genomes
GSEA	Gene Set Enrichment Analysis
WGCNA	Weighted gene co-expression network analysis
RT-qPCR	real-time fluorescence quantitative
DEGs	Differentially expressed genes
PPARg	peroxisome proliferator activated receptor gamma
AMPK	AMP-activated protein kinase
ULK1	unc-51 like kinase 1
mTOR	mechanistic target of rapamycin kinase
Lrg1	leucine-rich alpha-2-glycoprotein 1
PA	Palmitic acid sodium
TG	Triglyceride
TC	total cholesterol
ALT	Alanine Aminotransferase

AST	Aspartate Aminotransferase
BP	biological process
CC	cellular component
MF	molecular function
Lcn2	lipocalin 2
Steap4	STEAP family member 4
Hp	haptoglobin
Gnmt	glycine N-methyltransferase
Srebf1	sterol regulatory element binding transcription factor 1
Scd1	stearoyl-Coenzyme A desaturase 1
Fasn	fatty acid synthase
CRP	C-reactive protein
TNF-α	tumor necrosis factor-α
IL-6	interleukin-6
FABPs	fatty acid-binding proteins
C/EBPs	CCAAT enhancer-binding proteins
