# Supplementary material for: Intermittent fasting ameliorates MAFLD by downregulating Lrg1: insights from bulk RNA sequencing and functional verification
Source: Front Endocrinol (Lausanne). 2026 Feb 16;17:1754251. doi: 10.3389/fendo.2026.1754251 (PMC12950749; doi:10.3389/fendo.2026.1754251)
Supplement: Supplementary file 2 [file DataSheet2.pdf]

南方医科大学顺德医院 Shunde Hospital, Southern Medical University (The First People’s Hospital of Shunde Foshan) 实验动物伦理审查决议书

Decision of Laboratory animal ethics

决议编号/Resolution No.	SDYY-LH-12-2404-010	决议时间/Date of Resolution	2024 年 04 年 22 日
▲项目名称/Title of Project	超级增强子驱动 AHCY 高表达参与 HNF4α-PPARγ 调控网络在间歇性禁食改善 NAFLD 中的作用机制		
▲课题组负责人/Principal Investigator	梁华		
是否通过初审/Trial Result	☑ 通过 /Pass；　☐ 不通过 /Fail		
表决委员名单/The Committee Member List	曾小康、汤红峰		
表决形式/Voting Form	☐ 会议表决/conference vote；　☑通讯表决/ correspondence vote		
表决情况/Voting	有效表决人数/The number of valid voting：_2_人；<br>建议批准实验人数 /The number of approval voting：_2_人；<br>建议调整方案后批准实验人数 /The voting number of approval after revision：_0_人；<br>建议不予批准人数/The number of disapproval voting：_0_人。		
审查决议/Decision of Censor	经伦理委员会审查：Under investigation of Ethic Committee:<br>☑ 同意/Approval；　☐ 不同意/Disapproval；<br>☐ 修改后同意实施实验方案/Approval after revision。		
IACUC 主任或授权人签名 /Signature of IACUC Chief or Authorized Person	刘华<br>时间/Date: 2024年4月22日	南方医科大学顺德医院实验动物伦理委员会(章)/ Southern Medical University Experimental Animal Ethics Committee(stamp)	

注：标注“▲”由申请人填写。
