# Supplementary material for: Intermittent fasting ameliorates MAFLD by downregulating Lrg1: insights from bulk RNA sequencing and functional verification
Source: Front Endocrinol (Lausanne). 2026 Feb 16;17:1754251. doi: 10.3389/fendo.2026.1754251 (PMC12950749; doi:10.3389/fendo.2026.1754251)
Supplement: Supplementary file 3 [file DataSheet3.pdf]

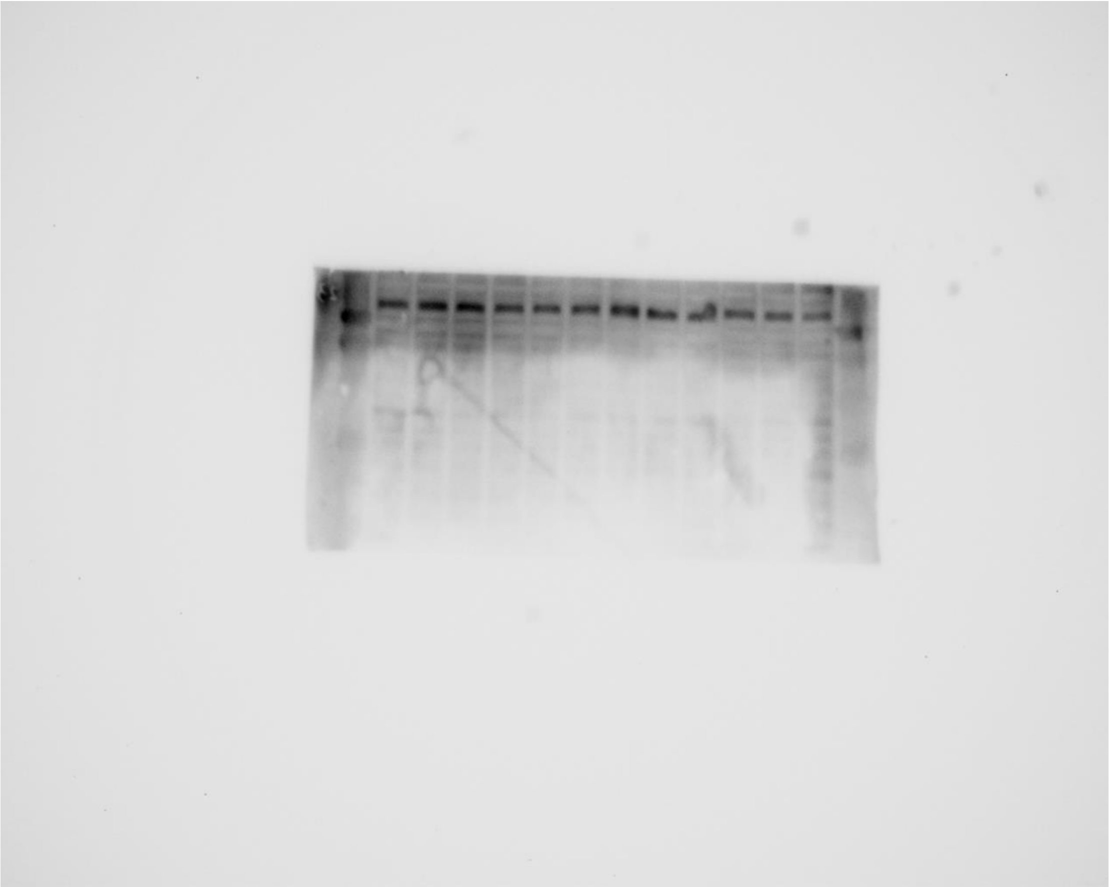

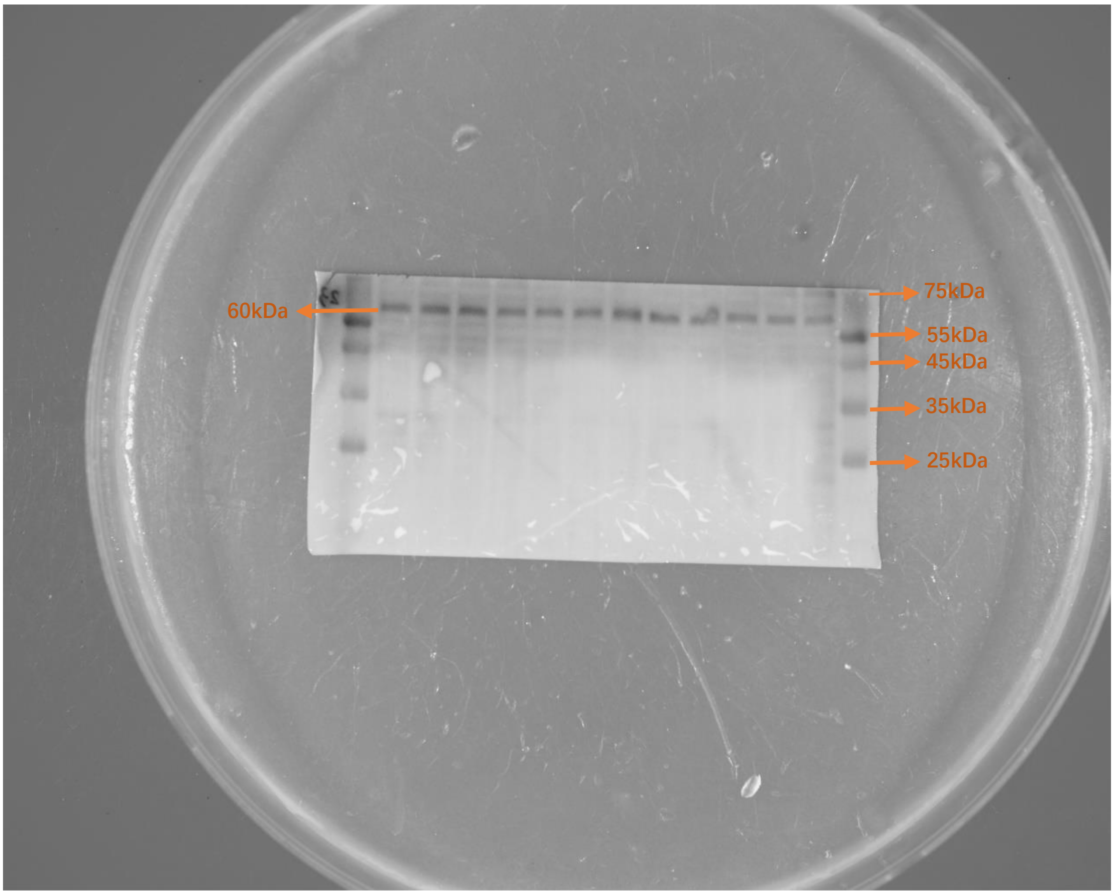
60kDa
75kDa
55kDa
45kDa
35kDa
25kDa

Supplementary Figure 1. The original picture of Figure 10A (p-AKT protein was detected by western blot).

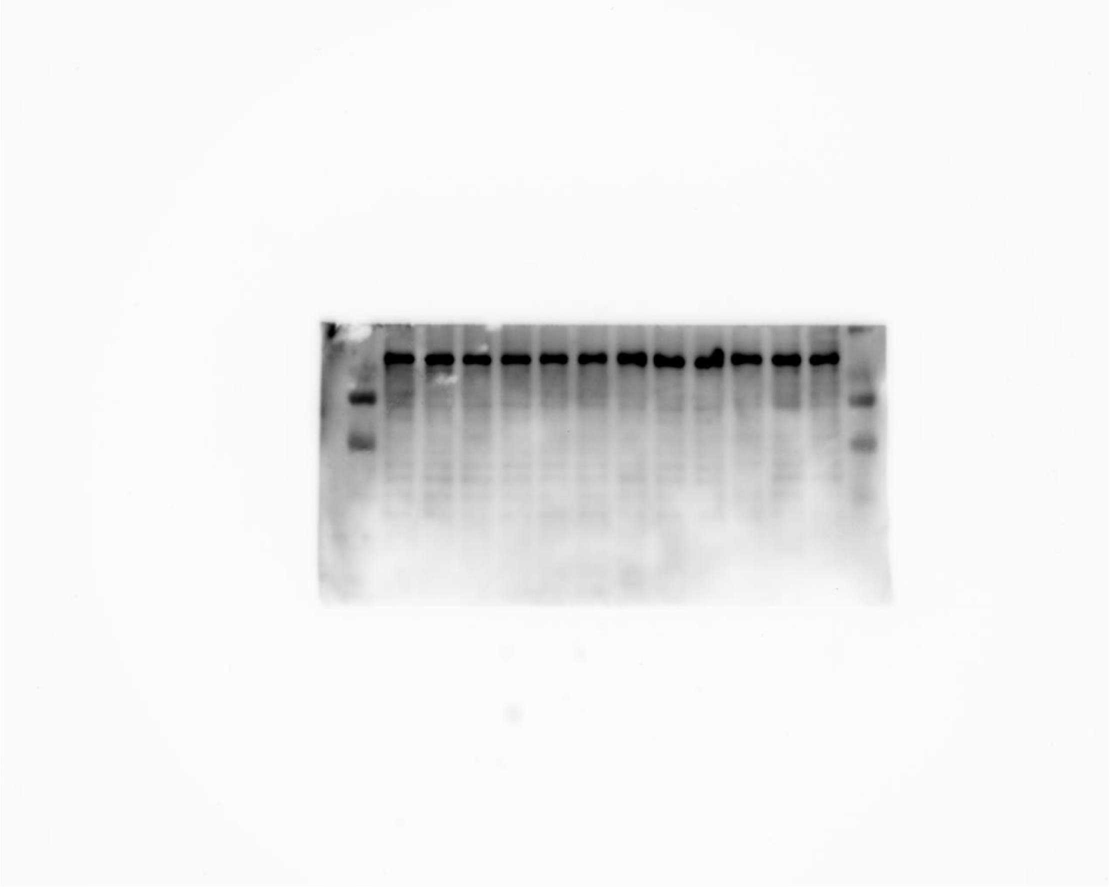

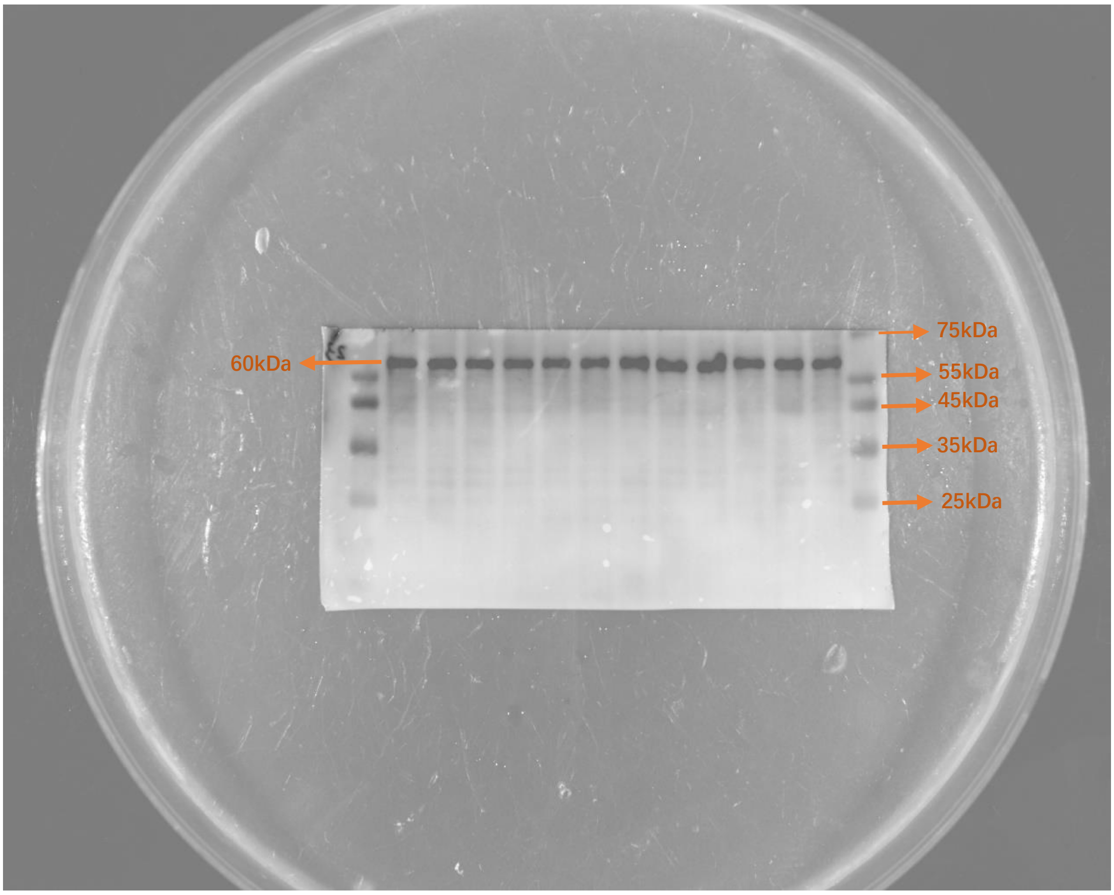

Supplementary Figure 2. The original picture of Figure 10A (AKT protein was detected by western blot).

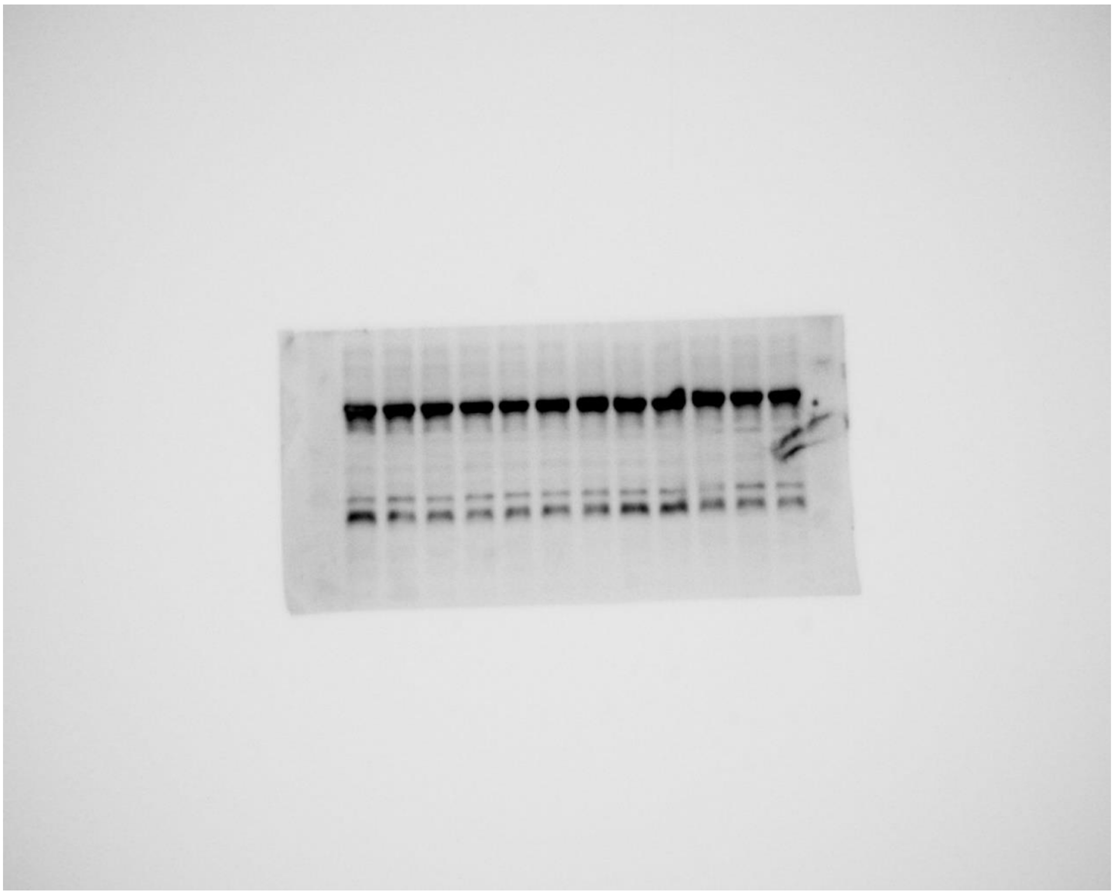

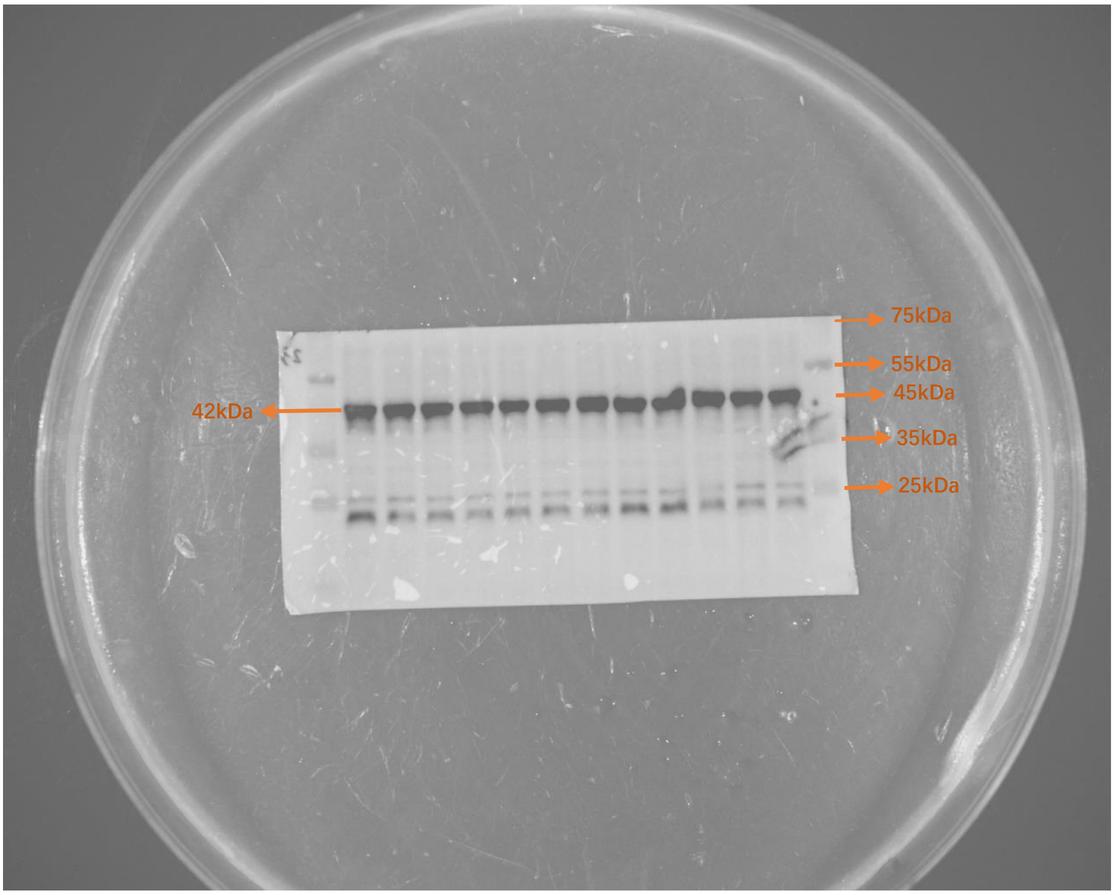
75kDa
55kDa
45kDa
42kDa
35kDa
25kDa

Supplementary Figure 3. The original picture of Figure 10A (β-actin protein was detected by western blot).

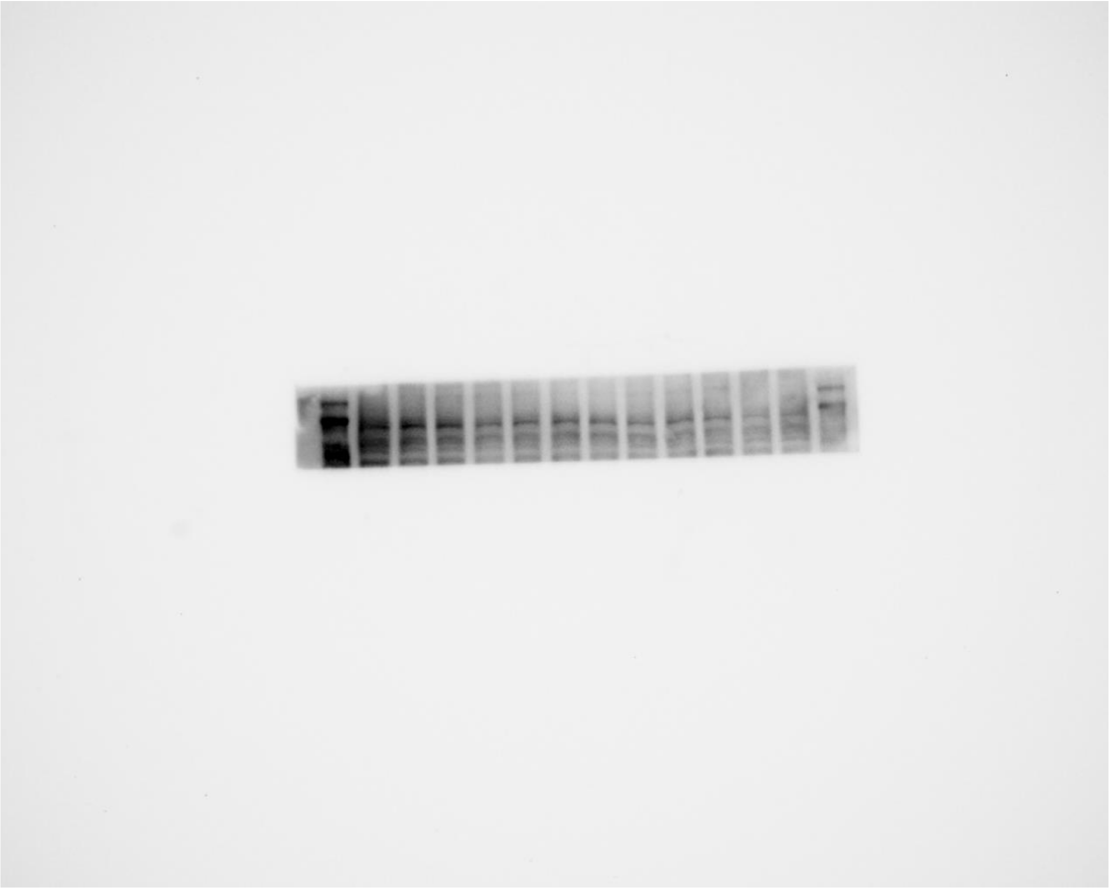

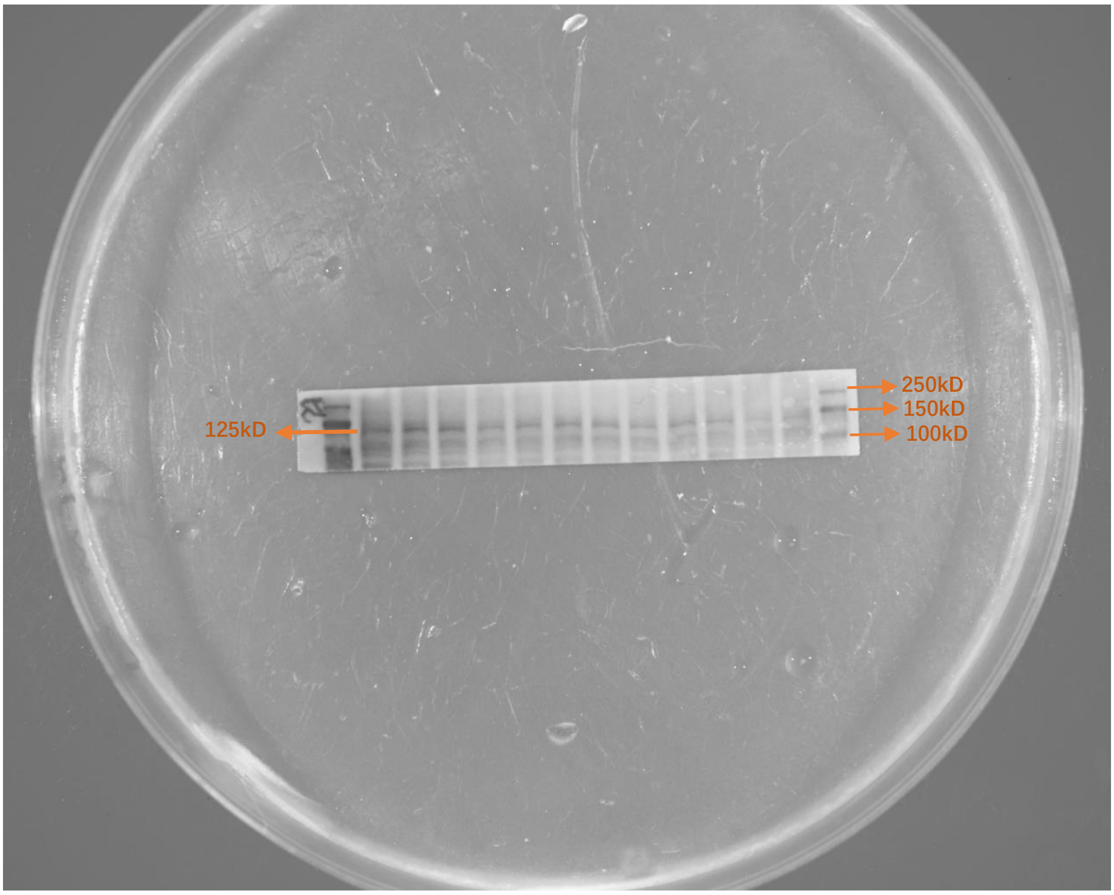

Supplementary Figure 4. The original picture of Figure 10A (Srebf1 protein was detected by western blot).

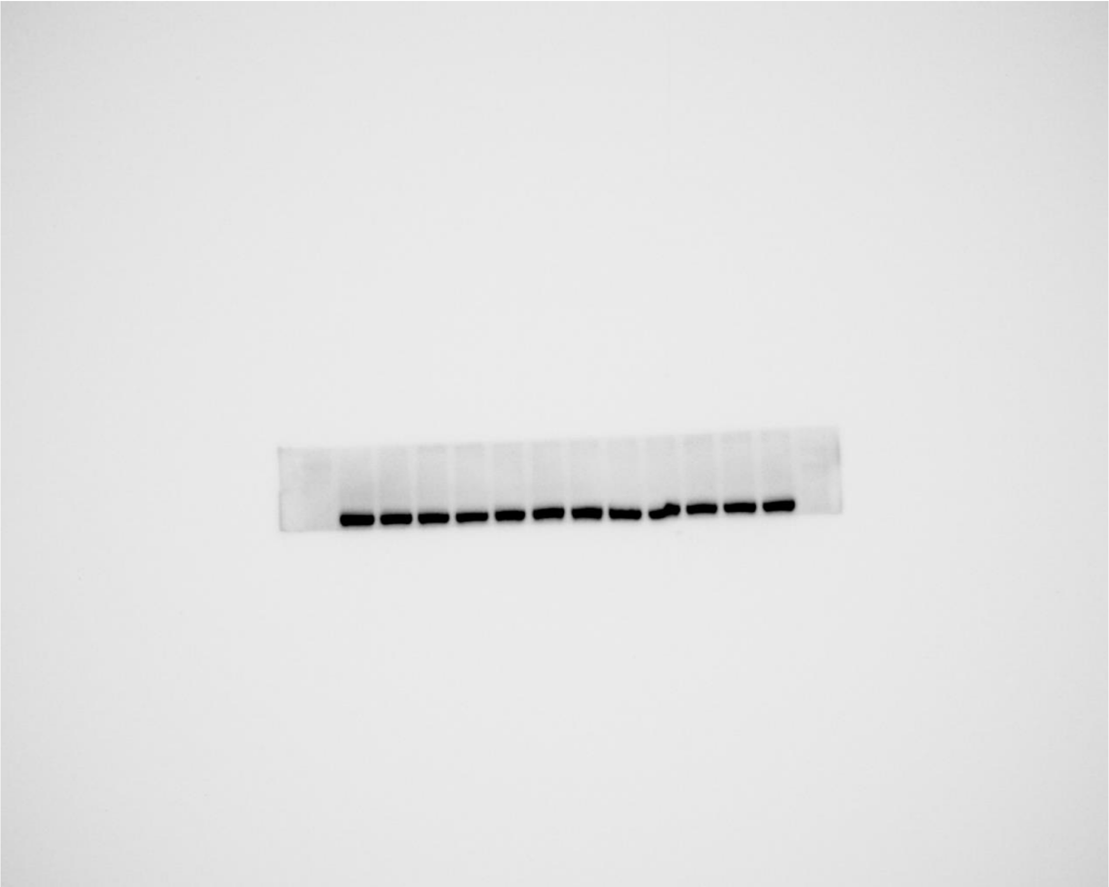

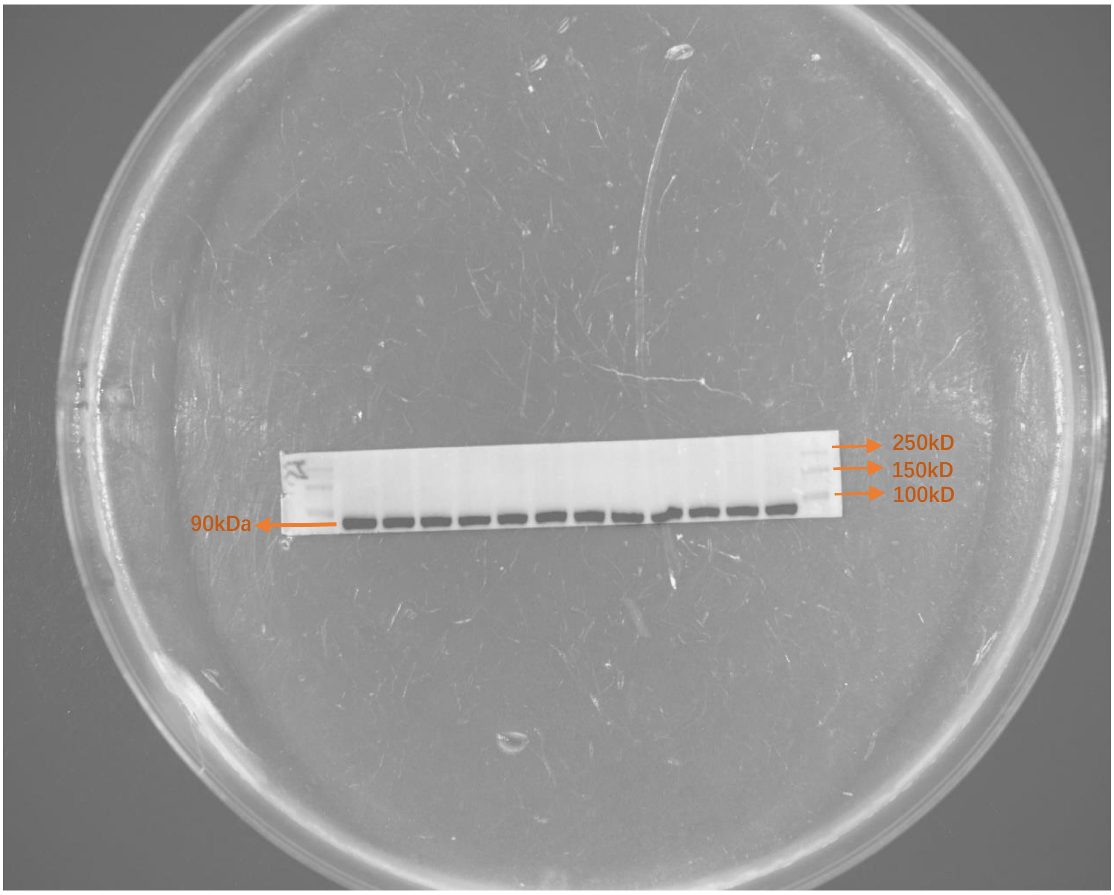
250kD
150kD
100kD
90kDa

Supplementary Figure 5. The original picture of Figure 10A (HSP90 protein was detected by western blot).

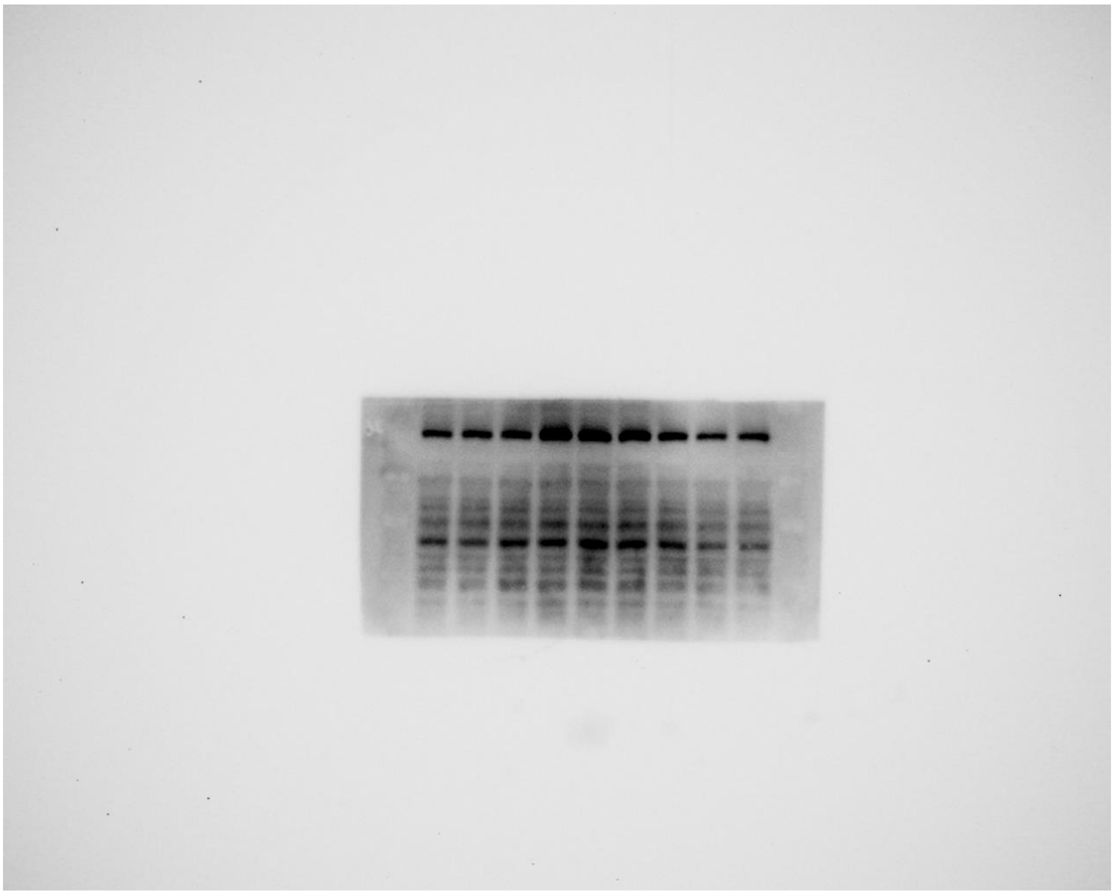

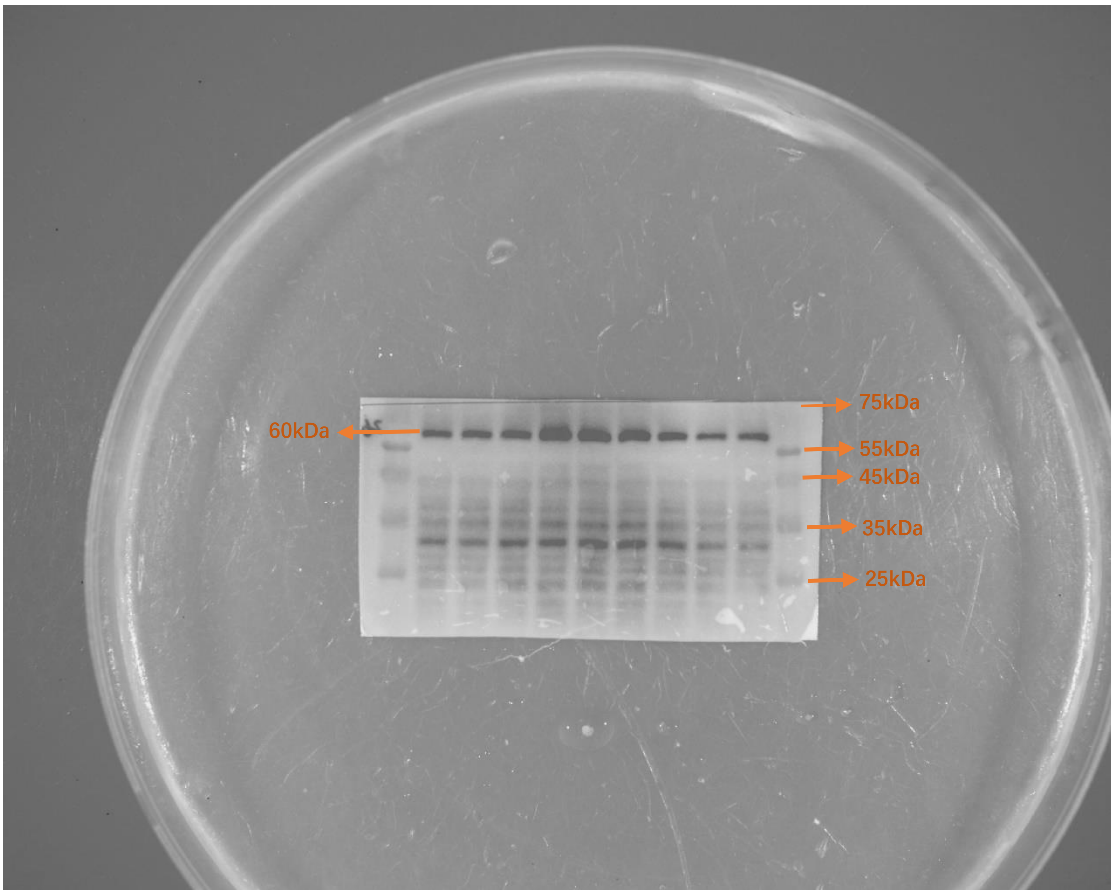

Supplementary Figure 6. The original picture of Figure 10G (p-AKT protein was detected by western blot).

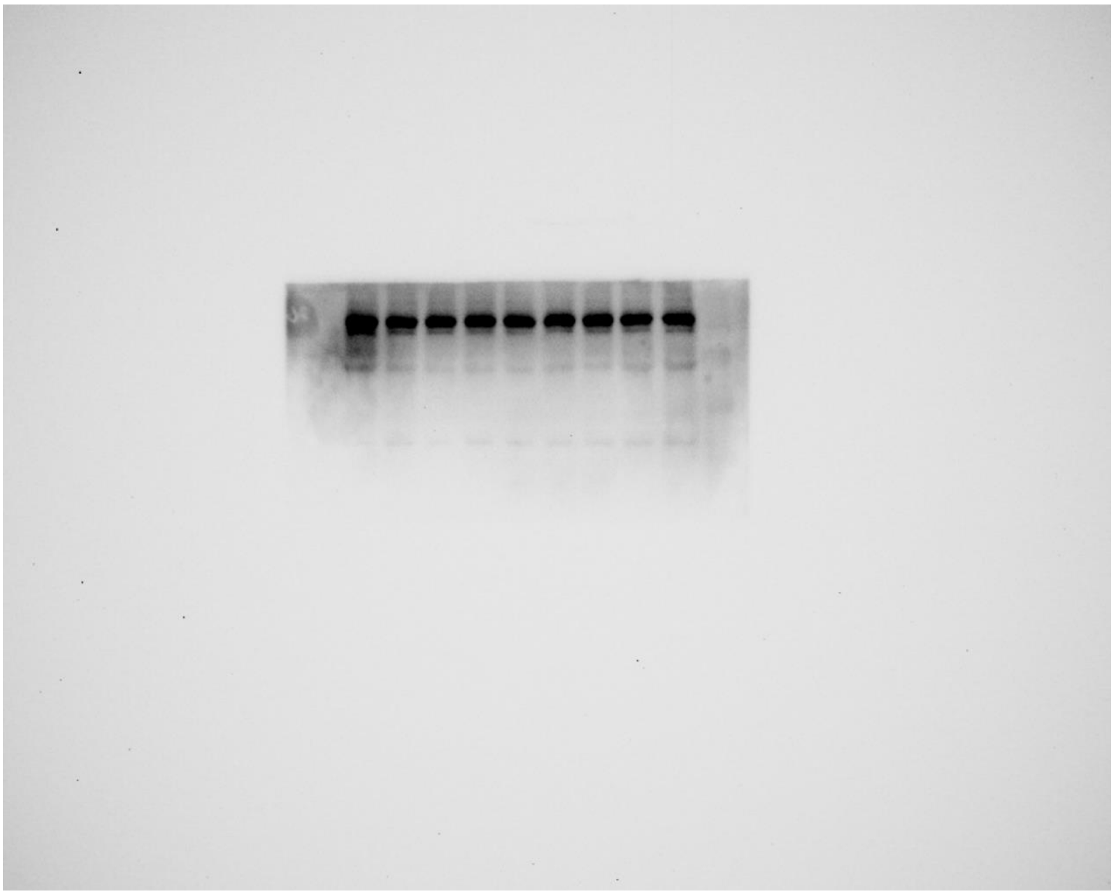

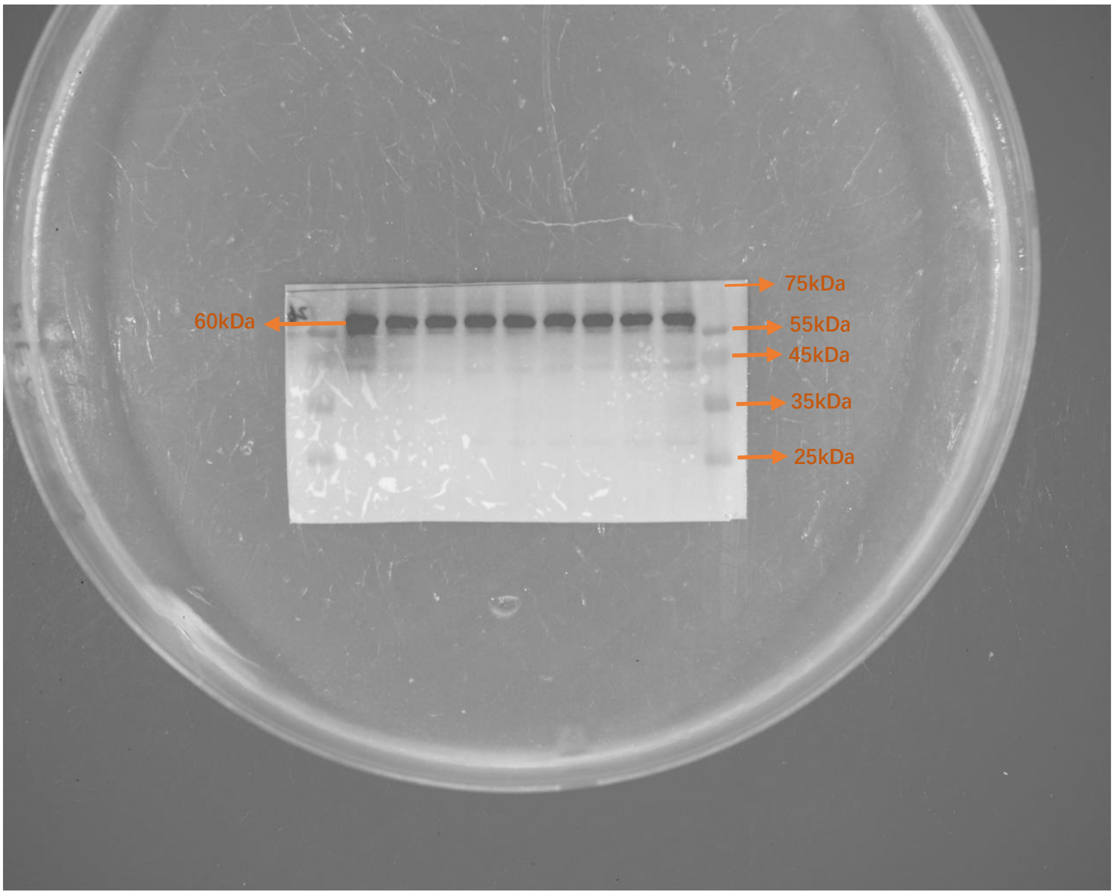
75kDa
60kDa
55kDa
45kDa
35kDa
25kDa

Supplementary Figure 7. The original picture of Figure 10G (AKT protein was detected by western blot).

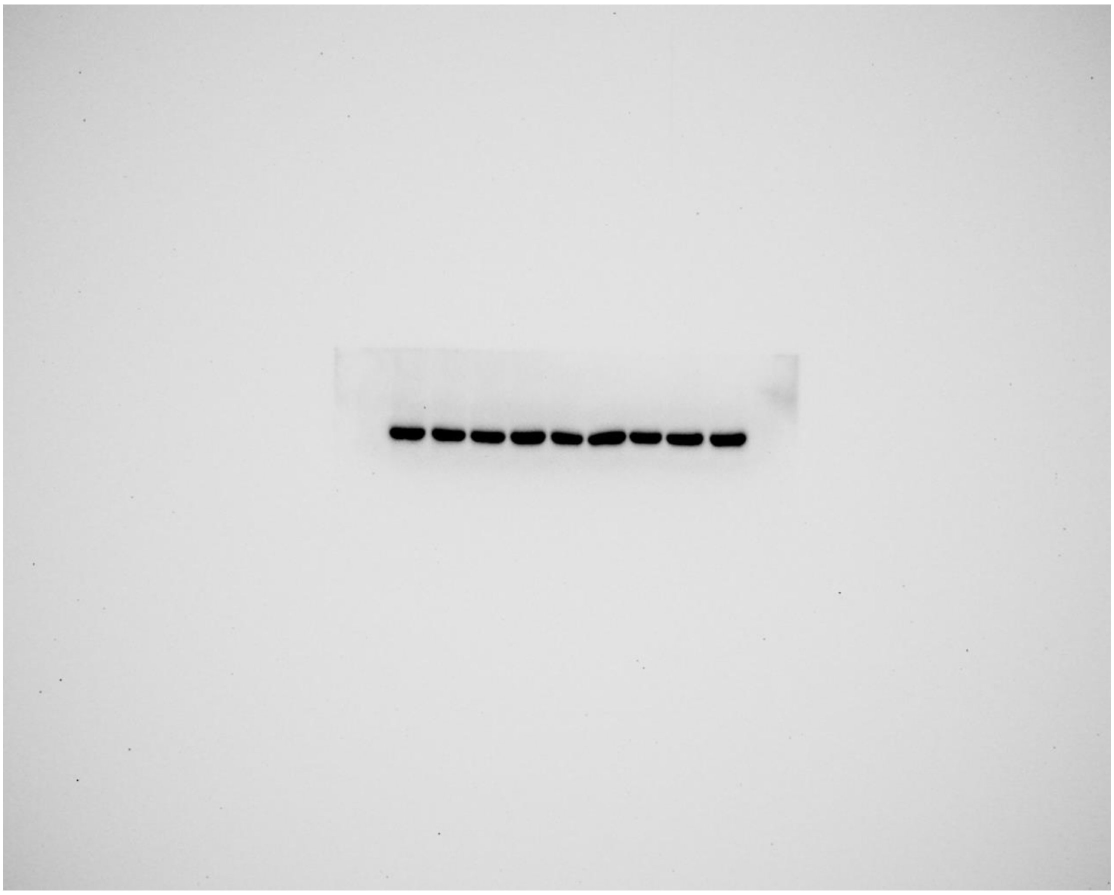

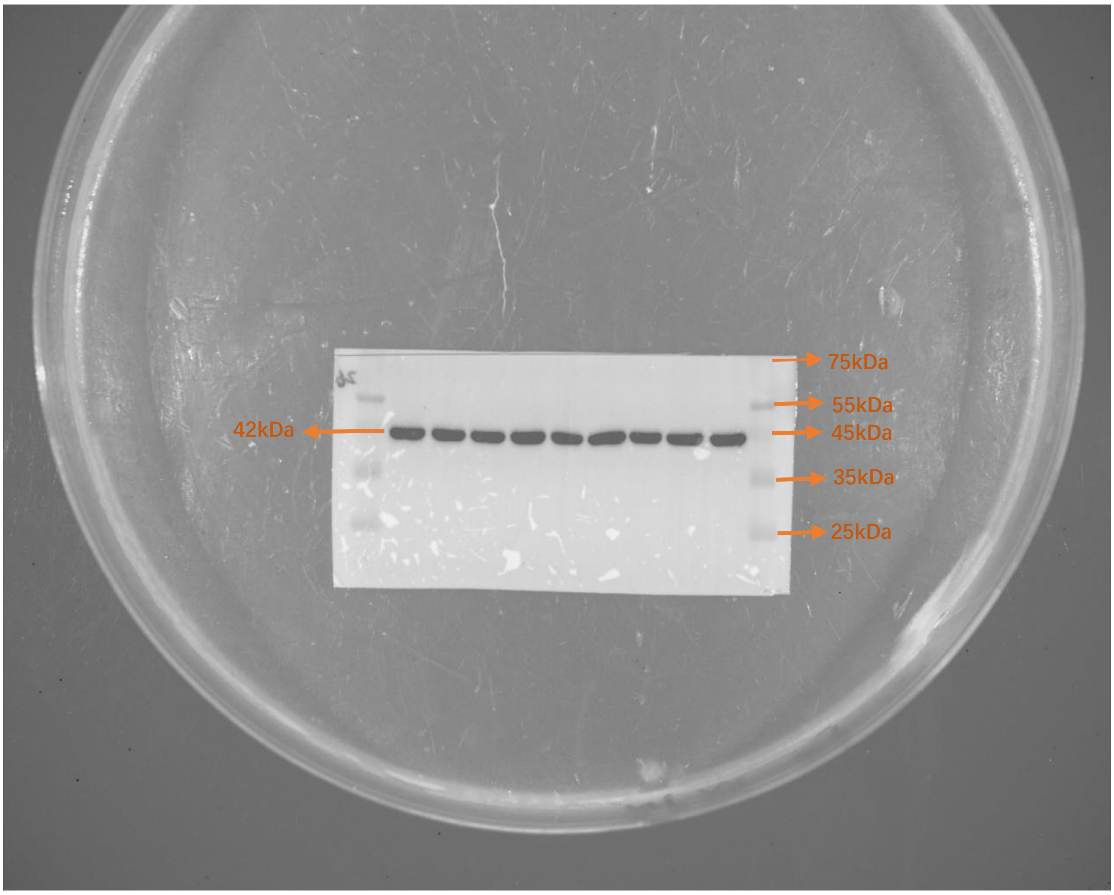

Supplementary Figure 8. The original picture of Figure 10G (β-actin protein was detected by western blot).

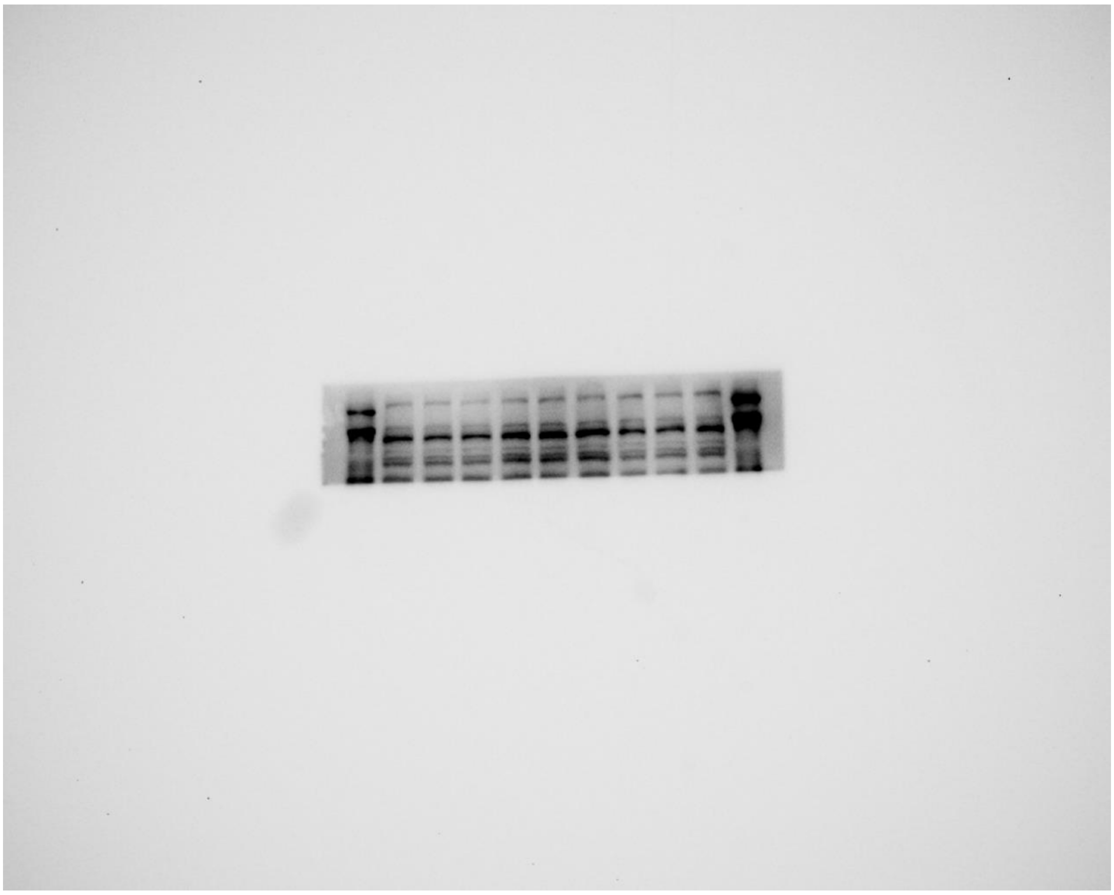

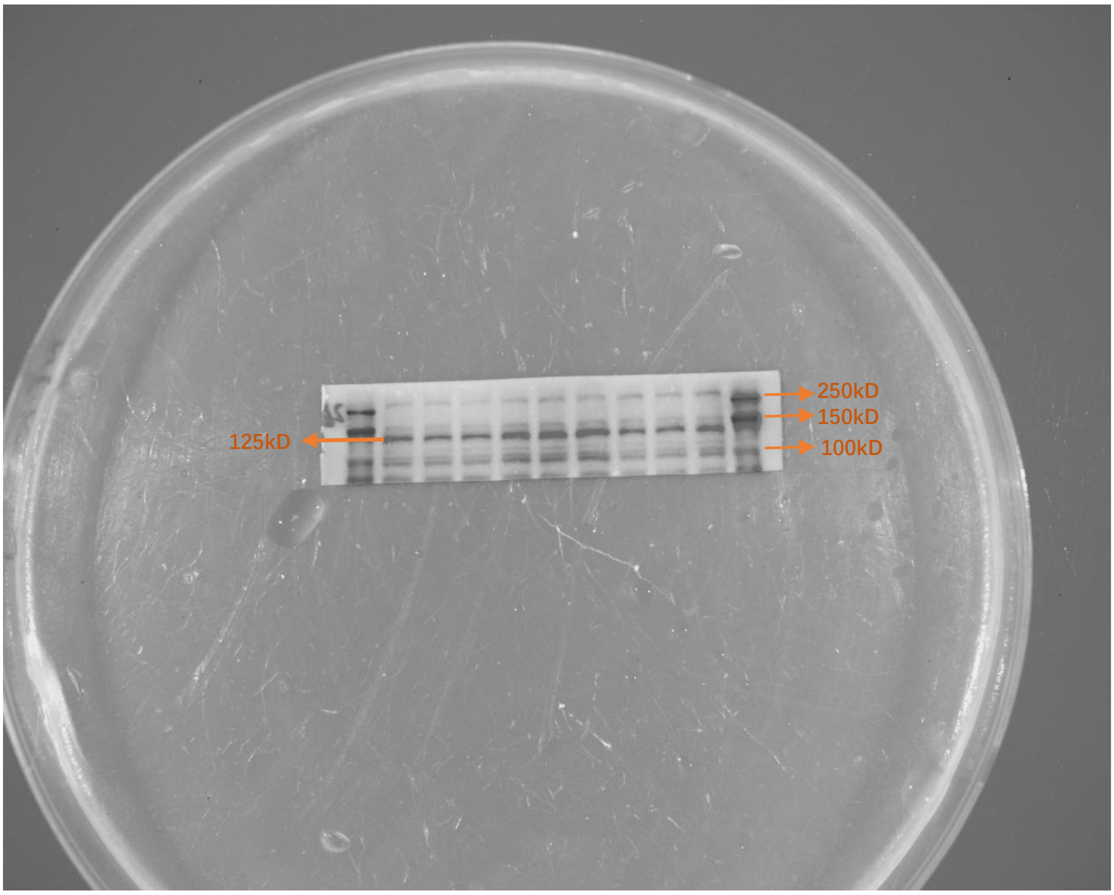
250kD
150kD
125kD
100kD

Supplementary Figure 9. The original picture of Figure 10G (Srebf1 protein was detected by western blot).

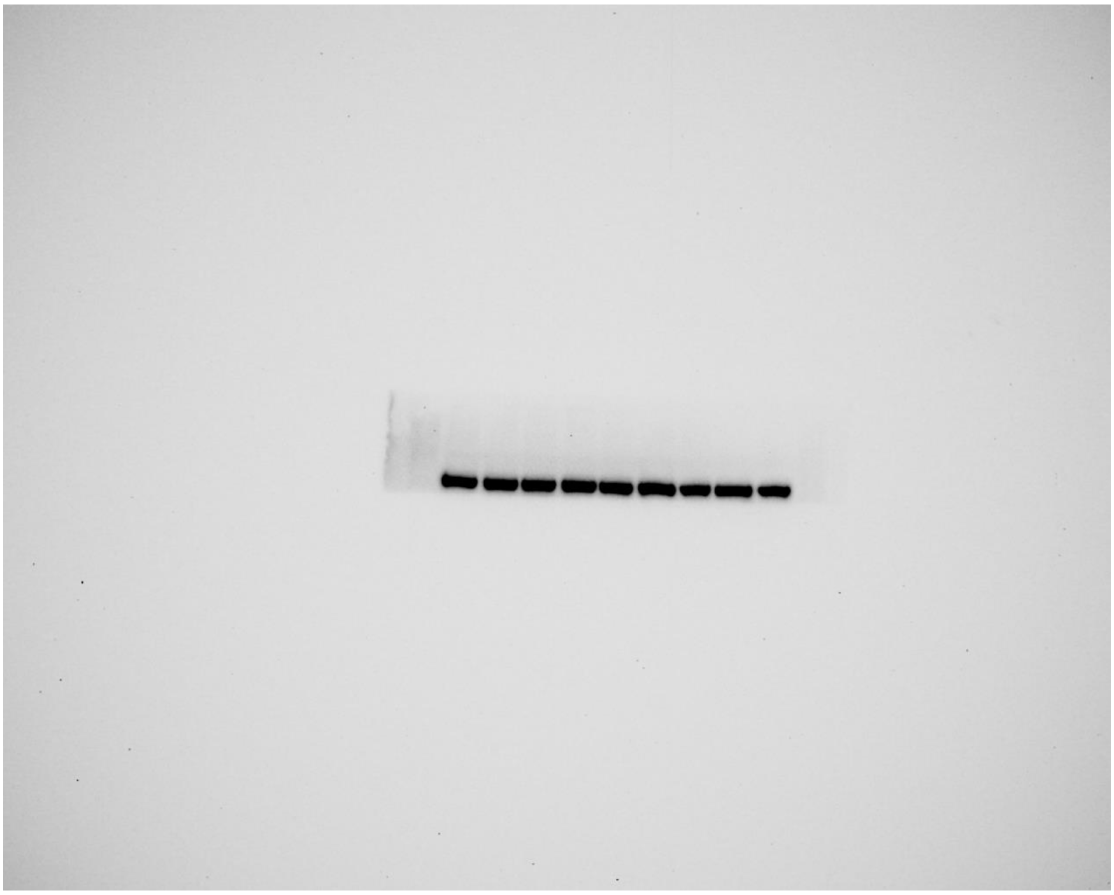

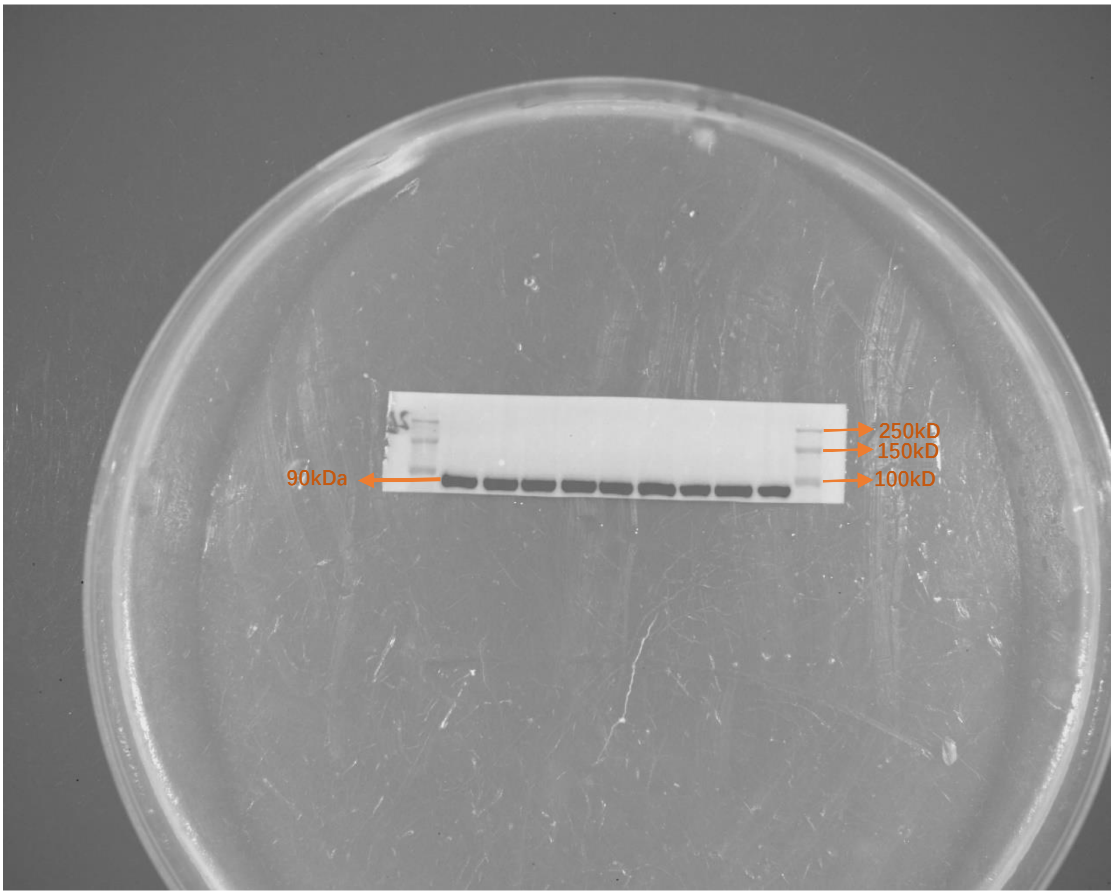

Supplementary Figure 10. The original picture of Figure 10G (HSP90 protein was detected by western blot).

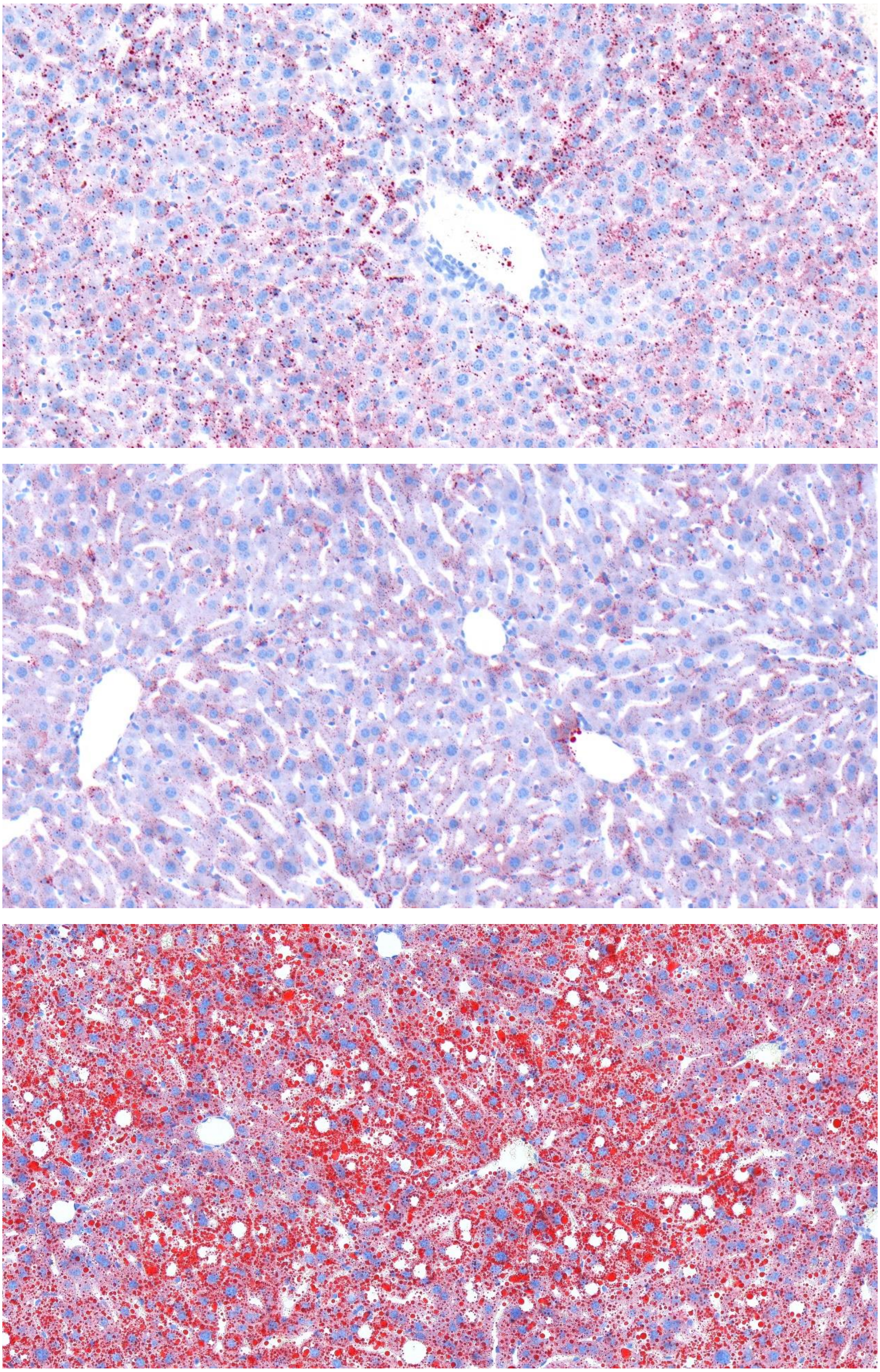

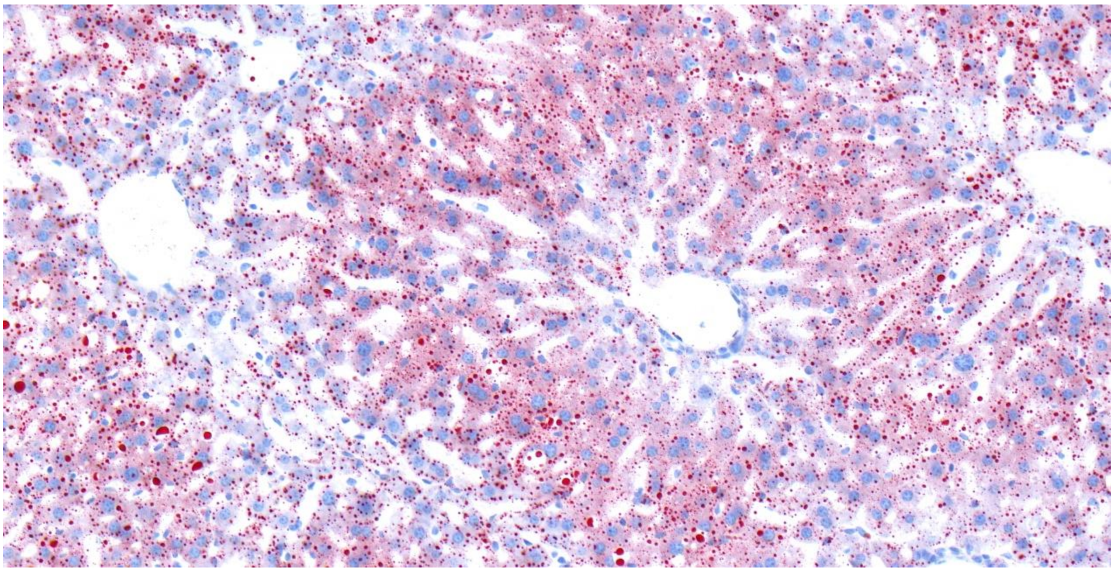

Supplementary Figure 11. Original image of Oil Red O staining in Figure 2A. The images from top to bottom represent the CD, iCD, HFD and iHFD group.

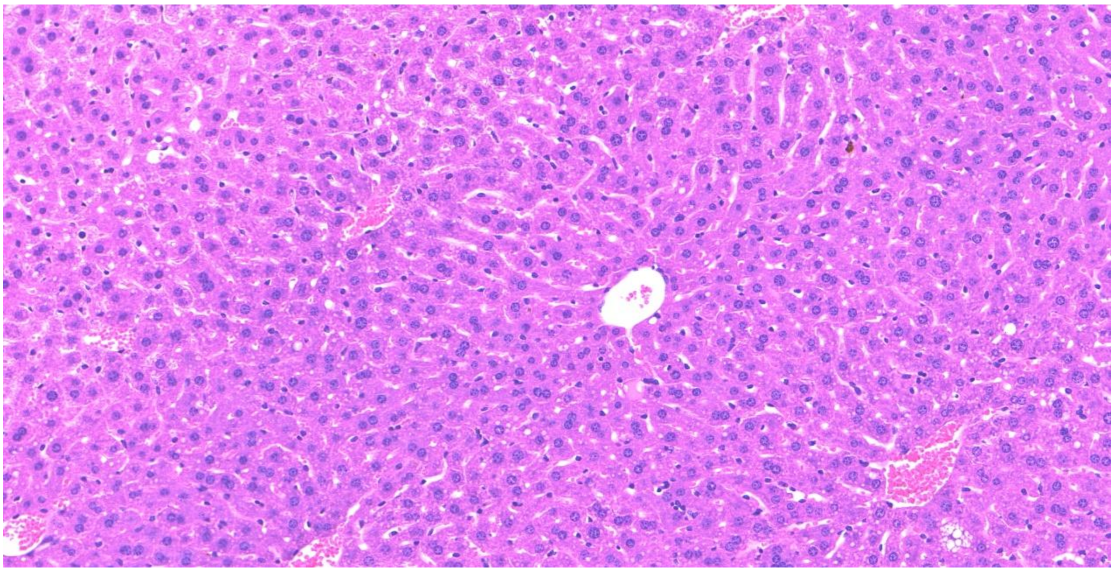

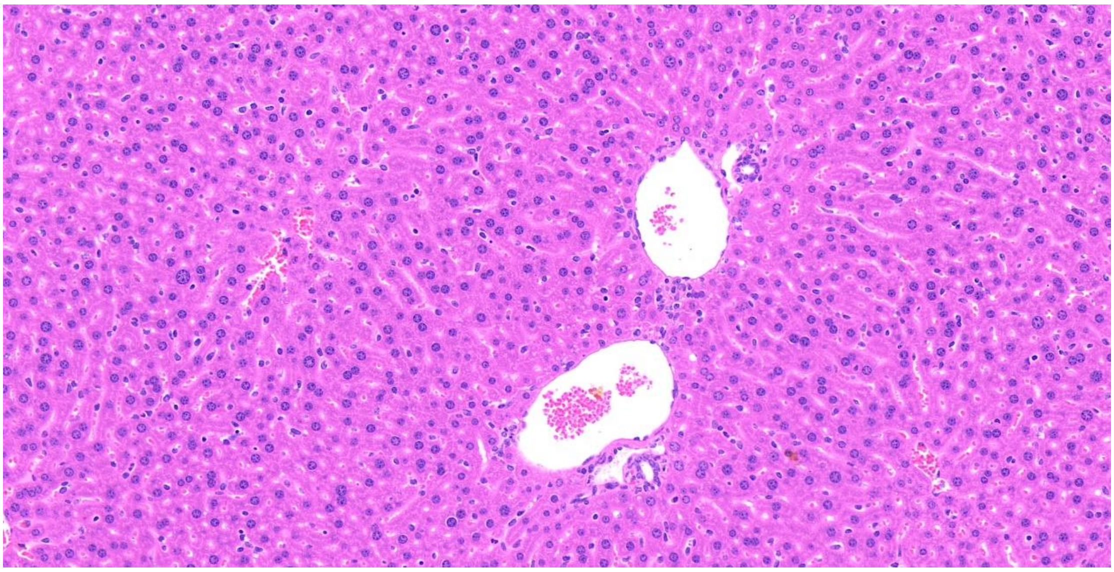

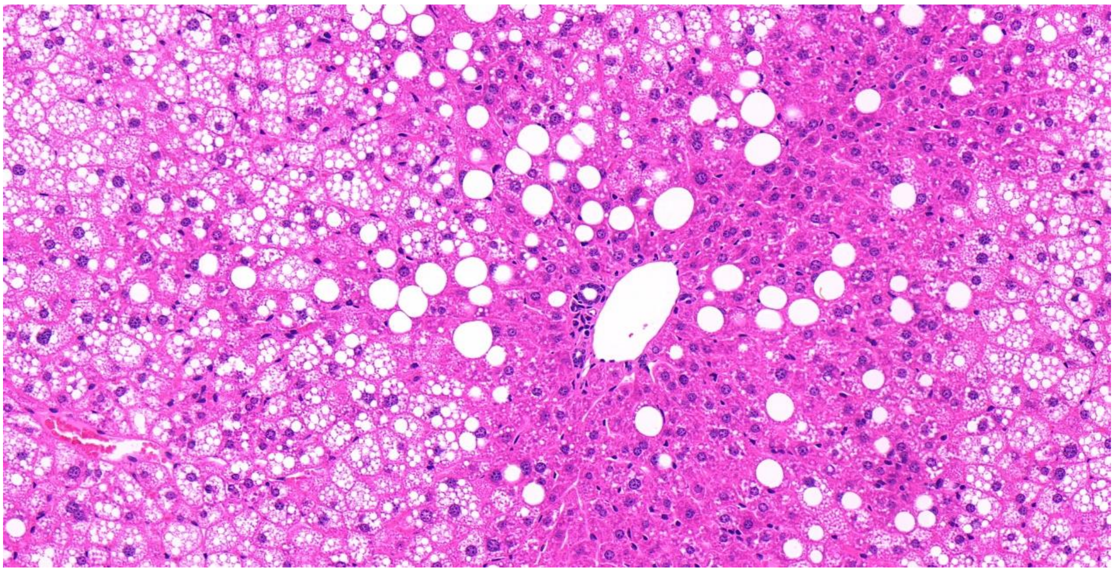

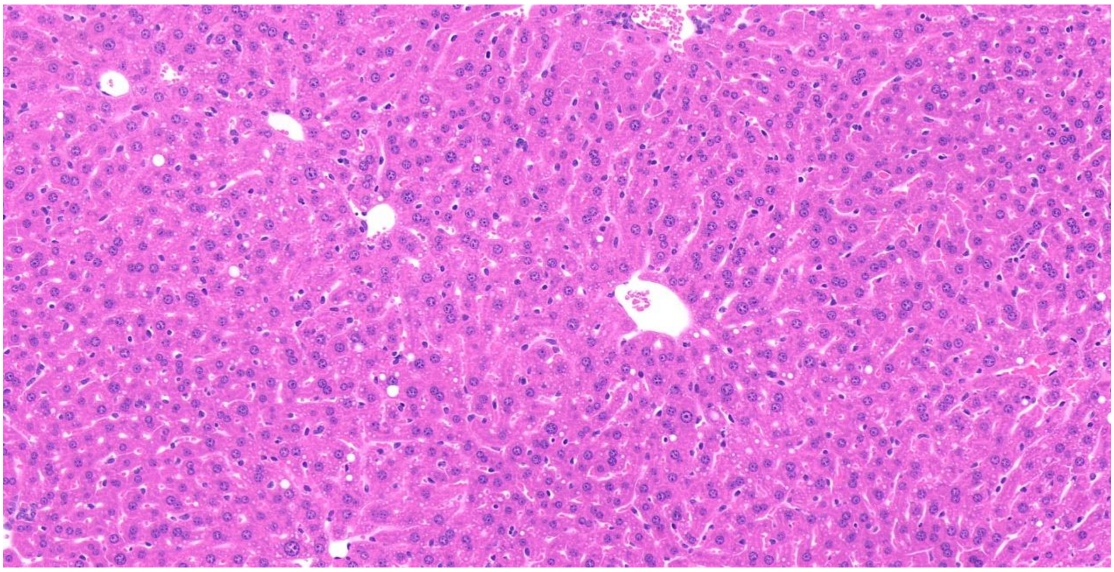

Supplementary Figure 12. Original image of HE staining in Figure 2A. The images from top to bottom represent the CD, iCD, HFD and iHFD group.

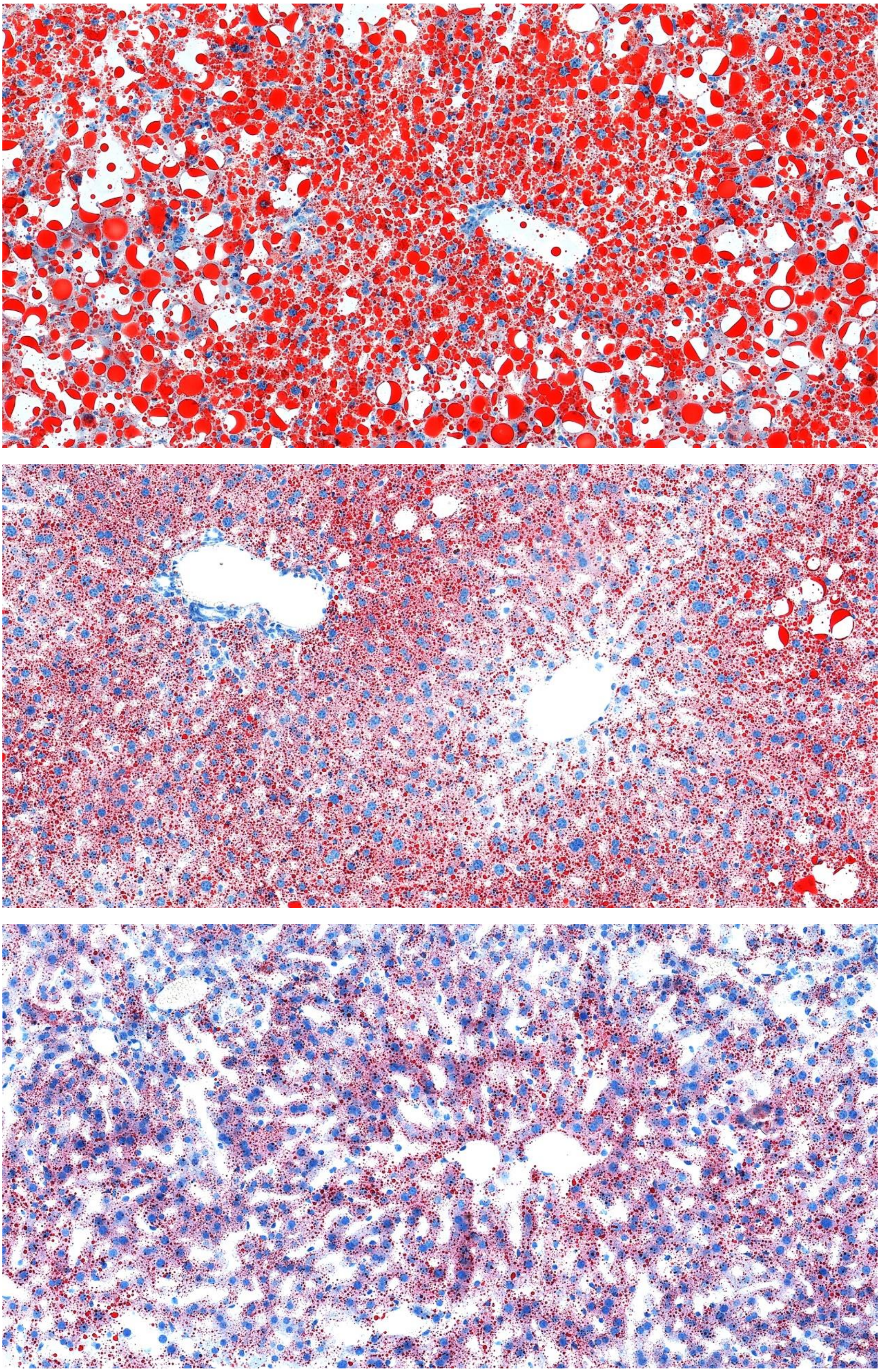

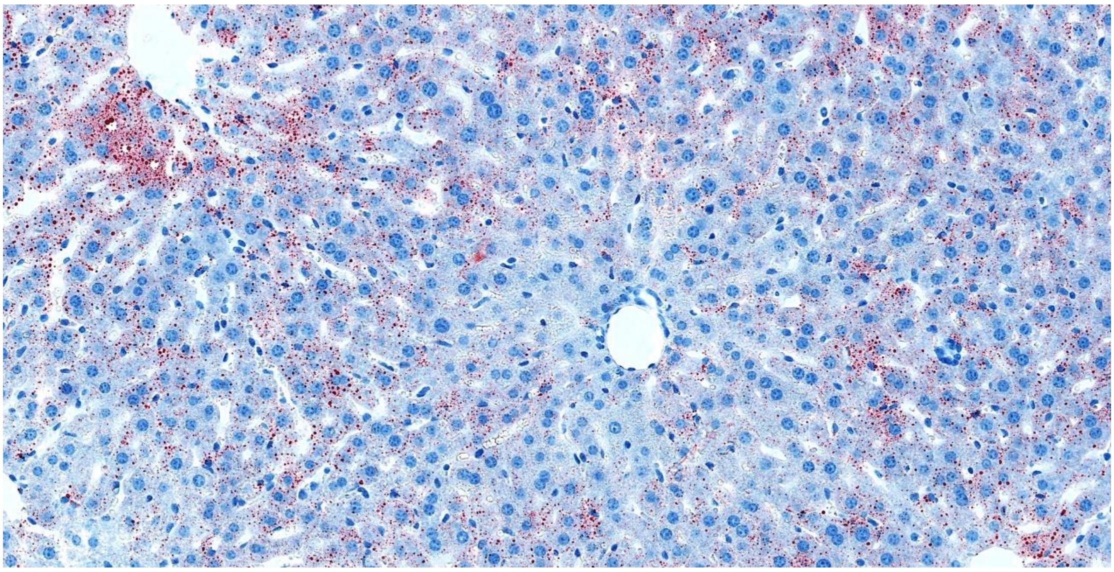

Supplementary Figure 13. Original image of Oil Red O staining in Figure 9A. The images from top to bottom represent the HFD+AAV-Ctrl, HFD+AAV-shLrg1, iHFD+AAV-Ctrl and iHFD+AAV-shLrg1 group.

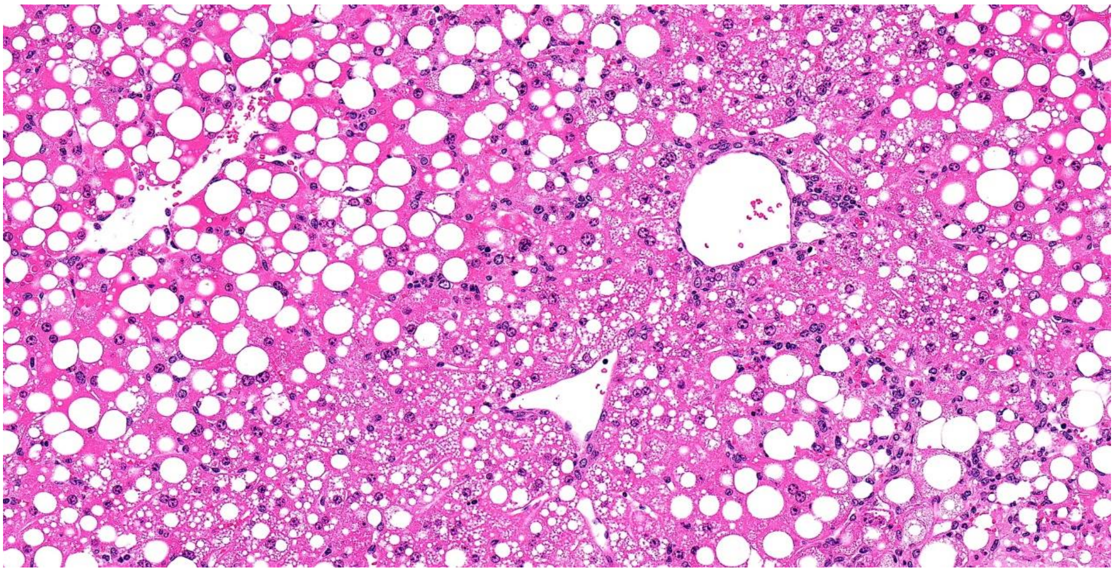

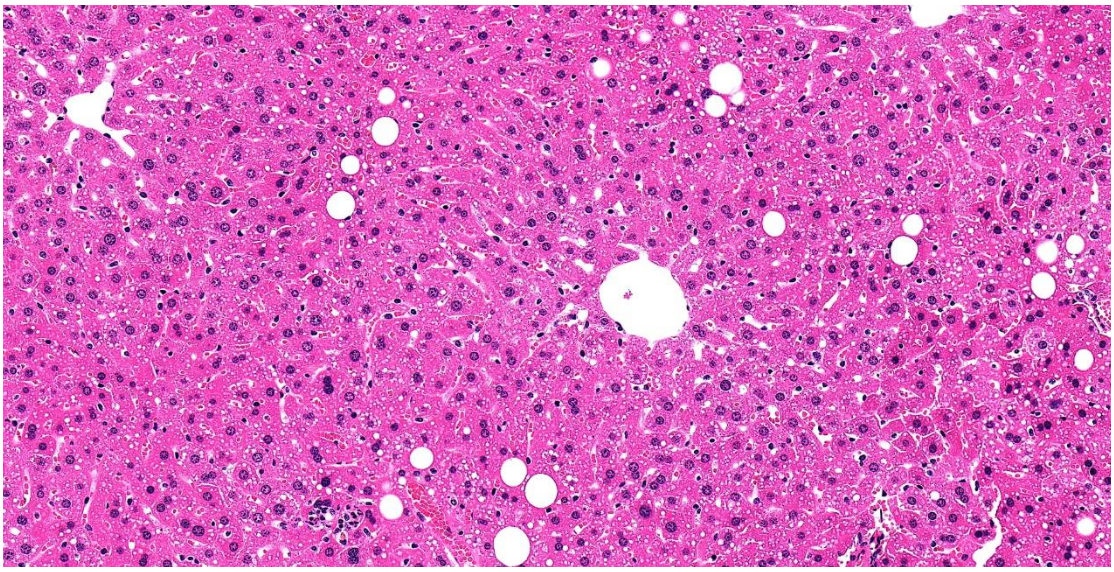

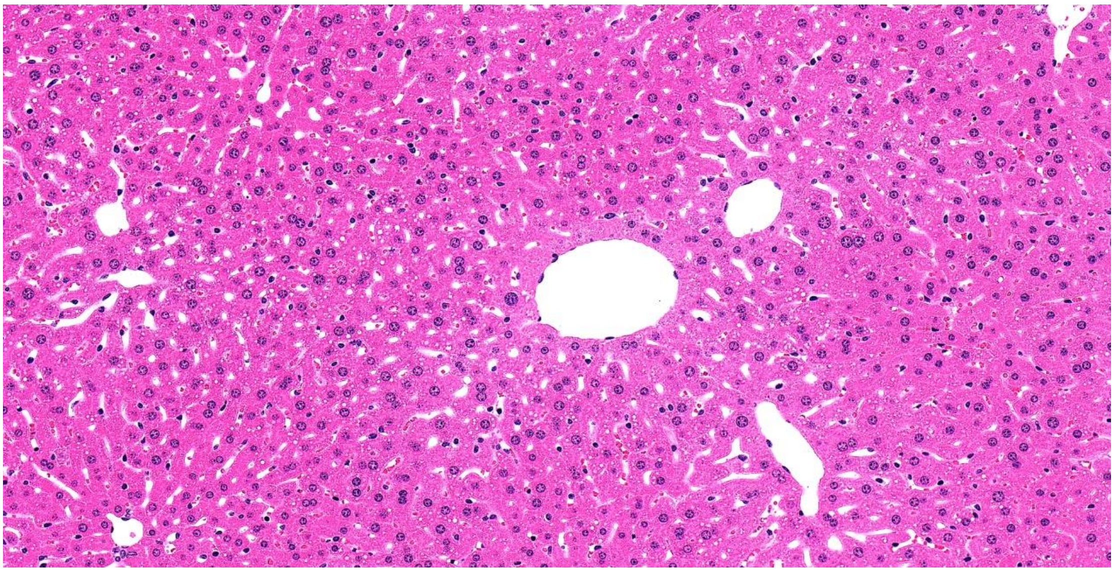

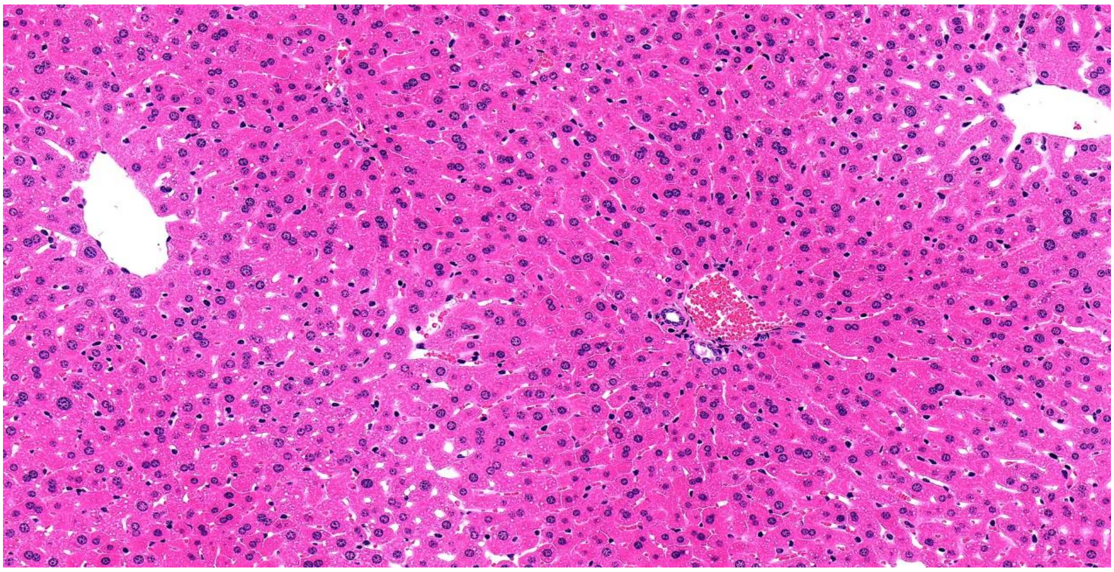

Supplementary Figure 14. Original image of HE staining in Figure 9A. The images from top to bottom represent the HFD+AAV-Ctrl, HFD+AAV-shLrg1, iHFD+AAV-Ctrl and iHFD+AAV-shLrg1 group.

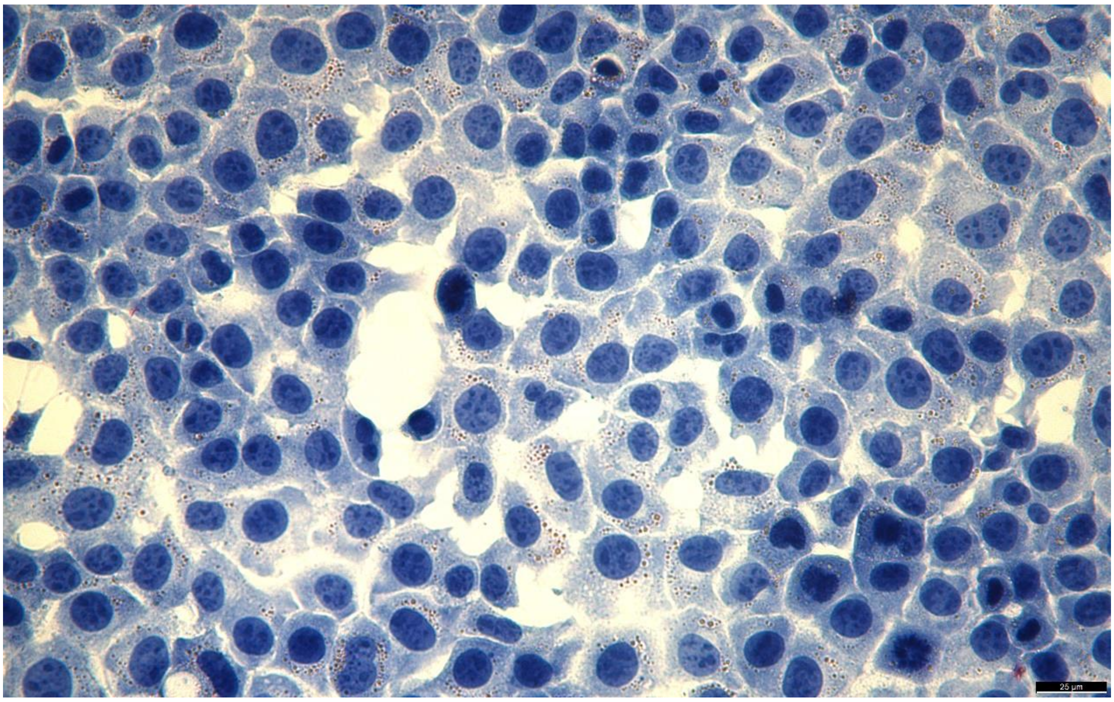
25 µm

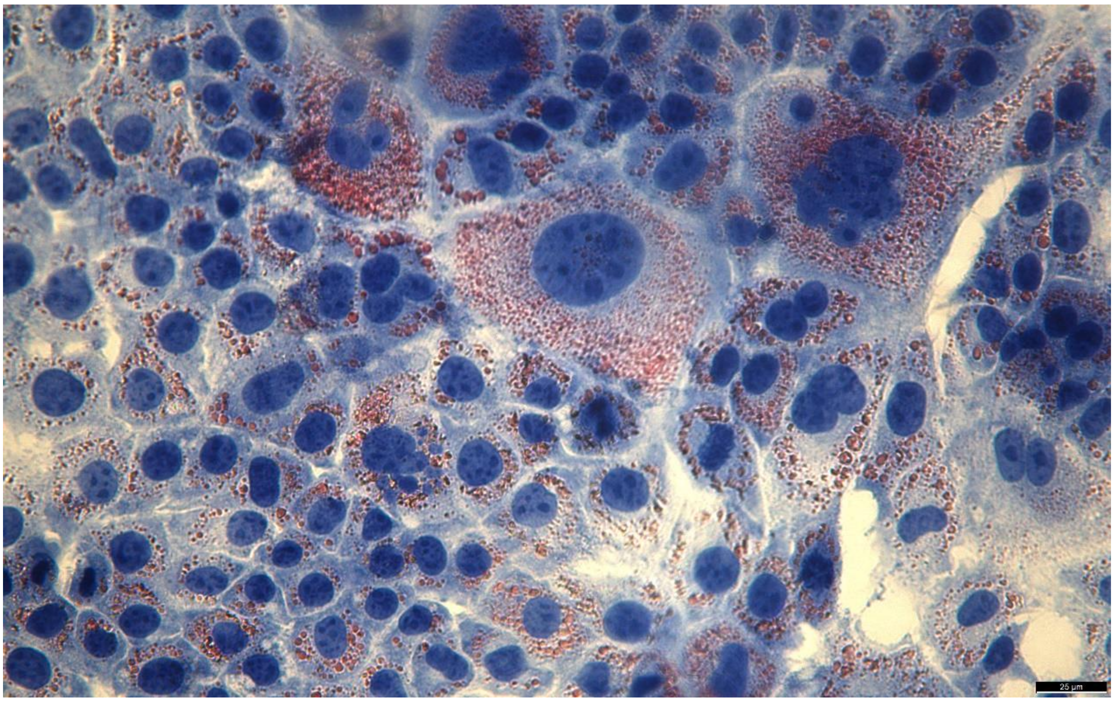
25 µm

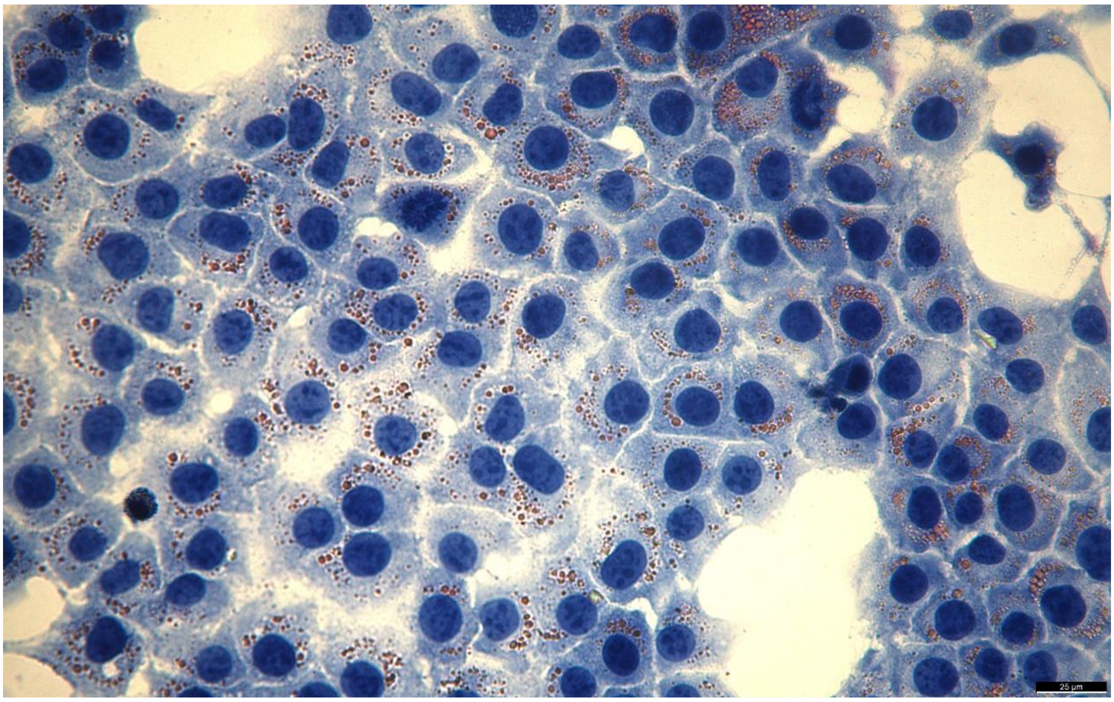

Supplementary Figure 15. Original image of Oil Red O staining in Figure 10C. The images from top to bottom represent the NC, PA and PA+siLrg1 group.
